# Real-Time Recognition of NZ Sign Language Alphabets by Optimal Use of Machine Learning

**DOI:** 10.3390/bioengineering12101068

**Published:** 2025-09-30

**Authors:** Mubashir Ali, Seyed Ebrahim Hosseini, Shahbaz Pervez, Muneer Ahmad

**Affiliations:** 1 Idexx Laboratories Inc., Auckland 4440, New Zealand; mubashir4ali@yahoo.com; 2 School of Information Technology, Whitecliffe College of Arts and Science, Auckland 1010, New Zealand; shahbazp@whitecliffe.ac.nz; 3 Department of Computer Science, University of Roehampton, London SW15 5PH, UK; muneer.ahmad@roehampton.ac.uk

**Keywords:** New Zealand Sign Language (NZSL), machine learning, k-Nearest Neighbours (KNN), AdaBoost (AB), computer vision, Support Vector Machine (SVM), Python, application, landmark, Random Forest (RF)

## Abstract

The acquisition of a person’s first language is one of their greatest accomplishments. Nevertheless, being fluent in sign language presents challenges for many deaf students who rely on it for communication. Effective communication is essential for both personal and professional interactions and is critical for community engagement. However, the lack of a mutually understood language can be a significant barrier. Estimates indicate that a large portion of New Zealand’s disability population is deaf, with an educational approach predominantly focused on oralism, emphasizing spoken language. This makes it essential to bridge the communication gap between the general public and individuals with speech difficulties. The aim of this project is to develop an application that systematically cycles through each letter and number in New Zealand Sign Language (NZSL), assessing the user’s proficiency. This research investigates various machine learning methods for hand gesture recognition, with a focus on landmark detection. In computer vision, identifying specific points on an object—such as distinct hand landmarks—is a standard approach for feature extraction. Evaluation of this system has been performed using machine learning techniques, including Random Forest (RF) Classifier, k-Nearest Neighbours (KNN), AdaBoost (AB), Naïve Bayes (NB), Support Vector Machine (SVM), Decision Trees (DT), and Logistic Regression (LR). The dataset used for model training and testing consists of approximately 100,000 hand gesture expressions, formatted into a CSV dataset for model training.

## 1. Introduction

Language serves as a fundamental medium for communication and social interaction. Language limitations, however, may restrict this effectiveness [1]. Different linguistic modalities, such as spoken language and New Zealand Sign Language (NZSL), can also make communication more difficult. Polite and transparent communication improves effectiveness and clarity, while impolite communication can cause a breakdown in a society and a lack of closeness and understanding.

**Figure 1:**
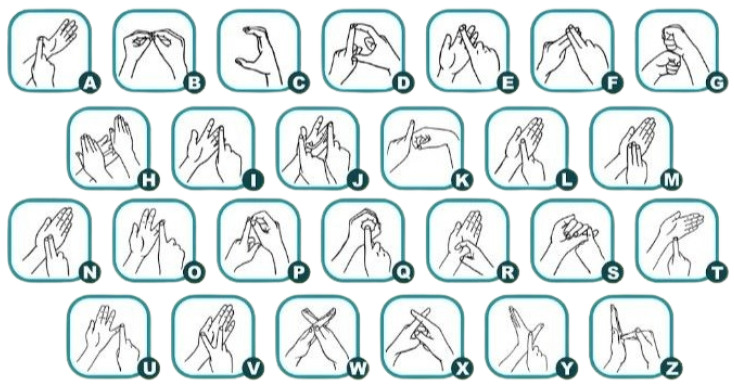
New Zealand Sign Language Alphabets [2].

In Figure 1, both the right and left hands can be used to make signs in New Zealand Sign Language (NZSL), and either hand can be the in charge hand. When fingerspelling, the dominant hand often plays the function of the pointer or pen and the non-dominant hand plays the role of the paper. To sign the vowels A, E, I, O, and U in NZSL, each digit is touched separately, beginning with the thumb. As with B, F, G, L, M, N, R, S, and W, the letters C, D, J, K, P, Q, T, V, X, and Y are made by forming the hand to resemble the letter. Specifically, only the lowercase versions are formed by the letters G, L, and R. There is no specific shape or relationship between the letters H and Z and their English alphabet counterparts. In addition, the audience can see some letters—like R and D—inverted.

**Figure 2:**
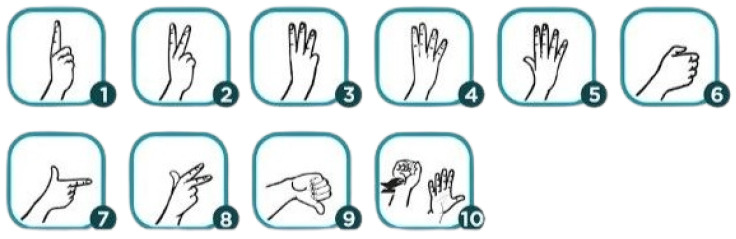
New Zealand Sign Language Numbers [2].

Figure 2 shows digits 1 through 5 are signed by extending the corresponding number of fingers, starting with the thumb for the number 1. For numbers 6 through 9, the fingers are positioned to represent each digit uniquely, with the number 10 often signed by making a fist and extending the thumb. Unlike some letters in the alphabet, all numbers have clear and distinct hand shapes in NZSL, making them easily recognizable. However, when signing certain numbers, like 9 and 6, the orientation of the hand may appear reversed to the viewer, depending on which hand is dominant. Additionally, some numbers may involve both hands, particularly when signing larger numbers or emphasizing a specific digit.

Intelligent, practical, and natural human-computer interaction is provided by hand gesture recognition. Sign language identification and gesture-based control systems are two important uses for hand gesture recognition technologies. Aim of the recognition system of sign language is to assist the deaf people connect with the hearing society more effectively by employing computer programs to automatically understand taught sign languages. Gesture-based human-computer interaction relies on the understanding of sign language, which is a highly structured and symbolic type of human gesture [3].

Recognizing individual alphabet gestures is the foundational step toward full word and sentence recognition in sign languages. However, there exists a significant semantic and structural gap between isolated letters and meaningful words or phrases. While alphabet gestures provide a linear, character-by-character translation method (finger spelling), natural sign language communication relies more heavily on holistic gestures, facial expressions, and spatial grammar. Therefore, developing a word recognition system requires not only accurate sequential letter detection but also handling temporal dependencies and contextual understanding, which are significantly more complex. This study focuses on high-fidelity alphabet recognition to build a robust foundation for future transition to full-word and phrase detection.

### 1.1. Novelty of the Research

The novelty of this research lies in the creation and utilization of a large-scale, dual-handed New Zealand Sign Language (NZSL) dataset tailored for gesture recognition using machine learning. Unlike prior works that primarily focus on single-handed alphabets or sign languages from other regions (such as ASL or BSL), this research addresses the lack of publicly available datasets for NZSL, which is inherently more complex due to its use of both hands. Furthermore, this study demonstrates the effective application of MediaPipe and OpenCV for precise landmark detection and integrates them with traditional ML algorithms to develop a real-time, high-accuracy NZSL recognition system. The dataset itself, comprising 100,000 annotated gesture instances, is a novel contribution that can be extended for future research in this underrepresented domain. This paper also provides open access to the source code to foster further research in NZSL recognition.

### 1.2. Background

Different linguistic modalities, such as spoken and sign language, can make communication more challenging. Many linguists have tried to define “language” since the middle of the 20th century, going beyond the traditional meaning and reconsidering aspects that were formerly thought to be fundamental. Language can be communicated visually and gesturally, even though it usually starts as speech and uses the vocal-auditory channel [4].

It’s a prevalent misperception that those who are deaf or hard of hearing only communicate through sign language. Sign language enables individuals to communicate emotions and ideas nonverbally. There are two kinds of gestures used in sign language: moving motions and still poses [5]. Hand gestures need to be recognised and deciphered for commands or interaction in a variety of contexts, such as virtual reality, robots, and sign language understanding [6]. The necessity for straightforward and impromptu communication in human-computer interaction has made hand signal recognition more crucial. Many techniques, such as deep learning-based methods, hybrid systems, and conventional computer vision techniques, have been used to recognise hand gestures [7].

New Zealand Sign Language (NZSL) has some similarities to British Sign Language (BSL) and Australian Sign Language (Auslan), it is sometimes referred to as “British, Australian, and New Zealand Sign Language” (BANZSL). NZSL and BSL are comparable in 62.5% of cases, however NZSL is similar in 33% of cases to American Sign Language (ASL). While sign languages like ASL and Pakistan Sign Language (PSL) utilise one-handed manual alphabets, NZSL, like BSL and auslan, uses a two-handed alphabet. NZSL is expressed via the signer’s hands, face, chest, eyes, and shoulders, just like many other sign languages [8].

**Figure 3:**
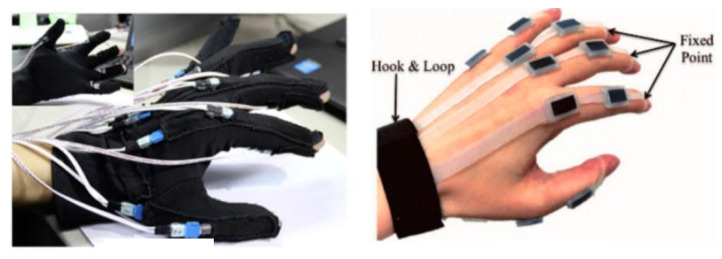
Sensor-Based Approach.

NZSL is an official language of New Zealand that is used by the Deaf community. NZSL users and the hearing community still face a substantial communication barrier despite its official status. The problem is made worse by the fact that hearing people are not generally proficient in NZSL and there are not enough certified interpreters. Ref. [9] reports that it is difficult to develop specialised credentials for translators in a variety of fields, including medicine, mental health, and education, due to existing infrastructure and supply issues. An estimated 23,000 people including 4000–5000 deaf persons use New Zealand Sign Language (NZSL) [10,11]. NZSL has a total of 26 alphabets, each represented by distinct hand shapes and movements. To build a communication system between the deaf and the hearing, sign languages must be interpreted into natural spoken languages [12]. New Zealand Sign Language alphabets are displayed clearly. Using sensors built into gloves intended for sign language communication is a further strategy. Despite the advantages these gloves provide, there are circumstances in which it might not be appropriate to use them [13] as shown in Figure 3.

Challenges arise in applications using reflective markers or sensor-based gloves [13], where hardware dependencies limit practicality. Vision-based systems like ours overcome these barriers by leveraging landmark detection, as seen in ASL translators [14]. However, NZSL’s dual-handed complexity exacerbates occlusion and tracking issues compared to single-handed languages, necessitating specialized solutions.

### 1.3. Problem Statement

The NZSL Act of 2006 legally recognised New Zealand Sign Language (NZSL) despite its declining usage. NZSL is becoming less widely used in conventional community settings, despite recent increases in institutional support. This trend is largely attributed to the increasing number of deaf children with cochlear implants who receive oral language instruction with minimal exposure to NZSL. Concerns are raised concerning NZSL’s long-term viability by this change [10]. While a number of machine learning algorithms have been studied for hand gesture recognition, such as SVM, KNN, RF, and MLP, a comprehensive assessment of their performance for NZSL recognition is absent. In order to create dependable and effective NZSL identification systems, it is imperative to evaluate these techniques, considering the potential impact of language decline on its preservation.

This study addresses the lack of accurate NZSL recognition systems by creating a 100,000-record dataset and evaluating multiple ML models to support automatic alphabet recognition. Subsequently, using various machine learning models and feeding them landmark distances from images, the objective is to create a system that can recognise NZSL automatically, which includes 26 letters. Additionally, the study aims to assess how well various machine learning models perform in the recognition of NZSL.

The project’s goal is to improve communication between New Zealand’s deaf and hearing communities by carefully evaluating the level of NZSL recognition technology today and utilising machine learning techniques.

### 1.4. Objectives

By utilising cutting-edge technical methods, the objective is to close the breakdown in communication between the deaf and hearing communities. places special emphasis on assessing the efficacy of various machine learning algorithms for precise sign identification.

- Generate a dataset of approximately 100,000 hand gestures, each annotated with distances between key hand landmarks to enable precise machine learning analysis, including thorough training and testing.
- Evaluate the efficacy of various machine learning models in NZSL recognition to determine the most effective strategy for enhancing communication between New Zealand’s deaf and hearing communities.
- Achieve an overall training accuracy of 90% or higher in NZSL alphabet and number recognition, with consistent performance in real-world settings, ensuring the system can reliably support effective communication.

This study investigated the use of AI and transfer learning in a New Zealand Sign Language translation method to enhance communication between the deaf community and those who are hard of hearing.

### 1.5. Significance of the Study

This study’s potential to close the communication gap between New Zealand’s hearing and deaf communities underscores its importance. By developing an advanced recognition system utilizing machine learning techniques, such as RF, KNN, and SVM, this research improves NZSL usability and accessibility. The innovative application facilitates real-time translation of sign language gestures into text, fostering greater inclusion and interaction between NZSL users and the general public.

Beyond general communication, this research has significant implications for digital health education and accessibility in healthcare settings. Deaf individuals often face challenges in accessing healthcare due to communication barriers with medical professionals who are not proficient in NZSL. The implementation of AI-powered sign language recognition tools in healthcare settings could enhance patient-provider interactions, enabling better diagnosis, treatment understanding, and patient engagement. This underscores the need for additional education and training in digital health solutions that incorporate AI-driven assistive technologies for healthcare professionals and caregivers.

By evaluating and refining different machine learning models, this research contributes to the ultimate goal of developing a robust, user-friendly tool that benefits both the deaf community and the general public. It also paves the way for AI-based digital health interventions, ensuring that healthcare services become more inclusive and accessible for the deaf and hard-of-hearing populations.

## 2. Literature Review

**Figure 4:**
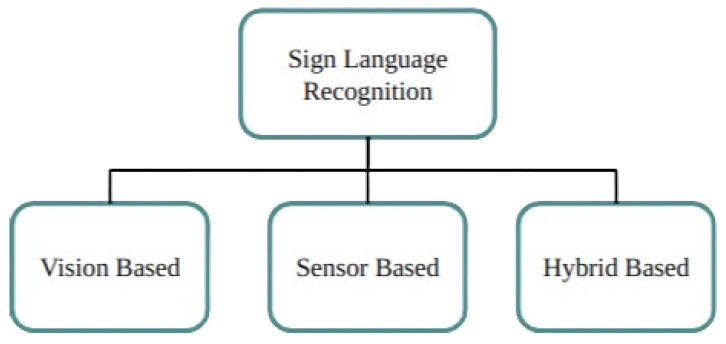
Sign Language Approaches [15].

Vision-based methods record video of sign language movements using cameras. In order to identify and decipher hand shapes, gestures, and face expressions, these systems analyse visual data. OpenCV and Mediapipe are two popular technologies used to extract and process information from video frames for real-time sign language recognition [15], see Figure 4. Sensor-based methods rely on wearable specialised sensors—such as wristbands or gloves—that are fitted with flex or motion sensors. These sensors take measurements of finger positions and hand movements, converting them into digital signals that the system can understand [15,16]. While this method can recognise gestures with great accuracy and detail, it frequently requires physical hardware. Hybrid-based strategies take advantage of the benefits of both vision-based and sensor-based techniques. These systems can improve recognition accuracy and resilience, supporting a variety of sign language motions and ambient situations, by combining visual data with sensor inputs. Hybrid systems seek to overcome the shortcomings of each distinct strategy in order to provide a more complete solution [17].

There exist two methods for the detection of sign language: vision-based and sensor-based [13,18]. Without the need for sensors or gloves, a vision-based system employs a camera to record hand motions as either static or dynamic images. The skin tone, lighting, background fluctuations, camera quality, and settings provide obstacles for this method, which is perfect for the routine lives of the mute and deaf [13].

**Figure 5:**
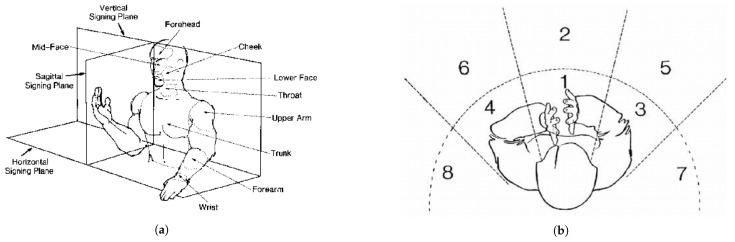
Different views of signing space. (**a**) The signing space and place of articulation signing parameter. (**b**) Areas of signing space (showing azimuth zones 1–8). (**a**) illustrates the place of articulation parameter. (**b**) shows areas of the signing space, with azimuth zones 1–8 corresponding to orientations around the signer’s body.

Boundaries that include the top of the head, the back, the breadth of the elbows, and the hips constitute the signing space. Different body points designate particular places for hand configurations, see Figure 5. Nevertheless, the distribution of these places is not uniform; most of the locations of opposing signs are centred on the face. According to Kyle and Woll, signs are typically articulated in close proximity to the face. Constraints pertaining to sign production and perception that affect how signs are received have an impact on this distribution [19].

Mathematical frameworks and algorithms are necessary to extract useful information from visual content, e.g., films and images. Computer vision methods are used here to recognise different aspects of the hand, like textures, contours, and landmarks. Following their extraction, these features are added to machine learning frameworks for additional examination and use [20]. CNNs and RNNs are commonly employed in DL frameworks to recognize hand signals, enabling the direct extraction of important features from input images. One approach utilizes 3D CNNs to capture both temporal and spatial information, enhancing hand signal recognition. Additionally, the temporal dynamics present in hand signals can be effectively represented using RNN architectures like Long Short-Term Memory (LSTM) networks [21,22].

Landmark detection is a crucial aspect of computer vision since it facilitates the recognition of hand gestures by pinpointing precise and informative spots on the hand. Traditional techniques like edge detection, the HOG, and the SIFT have been used to do this. But deep learning has made great strides, and deep learning-based algorithms are now preferred because of their improved capacity to decipher complicated images. One of the best examples of this trend is OpenPose, which effectively extracts landmarks from the hands, body, and face in the input image by using a multi-stage Convolutional Neural Network (CNN) [23,24,25].

Many machine learning approaches, such as DL models, support SVMs, KNN, and decision trees, have been successfully used to recognise hand gestures. K-NN is renowned for its ease of use and effectiveness; it operates by locating the input data point’s k-nearest neighbours. Decision trees are a popular technique that can handle both continuous and discrete data when it comes to hand motion identification. In the area of hand gesture identification, deep learning architectures—more specifically, CNNs and CNNs—have proven to be exceptionally effective [26,27,28].

Techniques of Computer Vision are used in recognising hand signal to recognise and extract important information from input photos. Hand signal identification techniques include edge detection, corner detection, HOG features, and SIFT. The goal of edge detection is to identify edges in the pictures so that the hand can be traced and its movements followed [29]. Corner detection uses the location and orientation of the hand’s corners in the photos to calculate its position. The look and feel of the hand in its immediate surroundings are captured by the feature extraction methods HOG, SIFT, and SURF. While SIFT and SURF use key spots in the image to construct descriptors for hand shape and texture, HOG features use histograms of orientated gradients to depict the hand’s direction and texture [29].

CV programming of Python (version 3.12.6) is a famous tool for merging ML and computer vision, especially for hand signal detection. In both domains, it makes the development of scalable and effective algorithms possible. A well-liked computer vision toolkit called OpenCV offers several features, such as object recognition, feature detection, and picture processing [29,30]. OpenCV uses feature extraction and computer vision techniques like edge and corner detection to identify hand signals. A variety of tools for classification, regression, and clustering in machine learning tasks are offered by Scikit-learn. Deep neural networks, including those for hand movement applications, can be created and optimised with TensorFlow’s comprehensive array of deep learning tools [31,32].

A recent survey reviewed over 50 studies on sign language recognition, concluding that combining visual hand data with motion and facial cues yields significantly better accuracy in low-context environments. This motivates future work on multimodal NZSL systems [33].

### 2.1. Related Work

YOLOv8 (You Only Look Once, version X) represents the latest advancements in real-time object detection, significantly outperforming its predecessors in terms of speed, accuracy, and flexibility. By integrating advanced backbone architectures like CSPDarknet and incorporating improvements such as PANet for feature aggregation, YOLOv8 enables more precise hand detection and gesture recognition in complex scenes. Unlike earlier models, YOLOv8 leverages anchor-free mechanisms and dynamic label assignment for enhanced training efficiency and generalization. This eliminates the need for handcrafted features and supports robust real-time landmark extraction across diverse lighting and background conditions, making it highly suitable for New Zealand Sign Language (NZSL) gesture recognition tasks [34].

Google’s MediaPipe Hands remains a leading approach for real-time hand landmark detection. Following research evaluated MediaPipe’s performance across various ethnic and hand shape datasets, showing its robustness in low-light and multi-hand conditions, reinforcing its suitability for dual-handed NZSL systems [35].

In the Journal of AI, ML and Neural Network, looks to be the source of the material you sent. The article describes a web application that translates sign language into spoken or written language and vice versa in order to improve communication between the deaf and mute community and people who can hear and speak. The system supports multiple sign languages, including ASL and ISL, and uses TensorFlow and MediaPipe’s Hand module for gesture recognition [14].

A combination of deep CNNs is used in the Journal of Electrical Engineering and Computer Science study to improve Arabic sign language recognition. It contrasts a multi-model method that concatenates these two networks with single models such as DenseNet121 and VGG16. Using a dataset of 220,000 static gesture photos, the study discovers that DenseNet121 and VGG16 together achieve near-perfect accuracy (100%) in both testing and validation stages, beating single models and other model combinations. By using complementing information from each CNN, our multi-model strategy performs better at identifying static Arabic sign language motions [5]. Table 1 provides a summary of the related literature.

### 2.2. Theoretical Framework

The quantitative analysis focuses on empirical data collection and analysis to assess the effectiveness and impact of sign language recognition systems. This involves gathering data from various sources, such as datasets obtaining from participants, statistical analyses, to assess how well sign language recognition systems are working. The quantitative analysis aims to measure the accuracy, efficiency, and user experience of recognition systems, providing objective insights into their effectiveness.

### 2.3. Gap(s)

Despite advancements in sign language recognition technology, significant gaps remain in addressing the specific needs of NZSL. A notable shortcoming is the lack of a standardized and effective gesture-based recognition system tailored for NZSL, particularly due to its reliance on dual-handed gestures. As opposed to one-handed sign languages, such as ASL, NZSL utilizes both hands to form letters and symbols, introducing additional complexity to recognition systems [36]. Existing technologies often excel with single-handed signs but struggle with the intricate hand movements and spatial relationships required for accurate dual-handed sign recognition [37].

Current research and commercial systems frequently focus on sign languages that use one-handed alphabets, such as ASL or Pakistan Sign Language. This bias creates a gap in technology when applied to NZSL, which involves more complex hand configurations. For instance, dual-handed signs in NZSL require the precise detection of both hands and their relative positions, a challenge that many systems have yet to address adequately. Additionally, there is a lack of comprehensive studies that evaluate the effectiveness of different algorithms for machine learning specifically for NZSL [38], leaving a gap in understanding which models are most effective for this language.

Furthermore, the limited availability of large, annotated datasets specific to NZSL exacerbates these challenges. Existing datasets often lack the diversity and scale needed to train robust machine learning models capable of handling the full range of NZSL signs. This limitation hinders the development of reliable recognition systems and impedes efforts to enhance communication between NZSL users and the hearing community.

## 3. Methodology

We methodically planned and executed a number of features to enable smooth exchange of messages among sign language users and others who depend on spoken language in order to bring the Sign Language Translation system to life. Two primary modalities comprise the implementation: Learning, New Zealand Sign Language Hand Gestures into Alphabets and Numbers. In addition, it includes study location, recruitment process, process analysis of data, ethical consideration.

### 3.1. Location

The study was conducted in Auckland, the largest and most populous city in New Zealand, known for its diverse cultural heritage and vibrant urban environment. Specifically, the research was carried out in collaboration with key locations, including Pukekohe Intermediate Park Side School, Buckland School, Tamaoho School, and the Auckland Deaf Society. These institutions provided an inclusive and supportive environment for the collection of hand gesture data. Auckland spans an area of 1086 km^2^ and, as of 2023, is home to over 1.7 million residents, with a significant proportion living in urban settings. This setting offered a rich demographic and cultural context, essential for the successful execution of this New Zealand Sign Language (NZSL) study.

### 3.2. Inclusion Criteria

**Participants must meet one or more of the following criteria:**

- **Teachers**: Actively teaching or with experience in teaching.
- **Trained Individuals in NZSL**: Participants with formal training or certification in New Zealand Sign Language (NZSL).
- **Special School Teachers**: Educators working in special schools, particularly those interacting with deaf or hearing-impaired students.
- **Instructors with Some Knowledge of NZSL**: Individuals who may not be formally certified but have working knowledge or experience using NZSL in instructional settings.

### 3.3. Exclusion Criteria

**Participants who do not meet the above criteria, including:**

- Individuals with no prior teaching experience.
- Persons without any formal or informal knowledge of NZSL.
- General educators not involved in special education or NZSL instruction.
- Individuals lacking practical exposure to NZSL in any professional or instructional context.
- Persons working outside the education sector, regardless of their familiarity with NZSL.

### 3.4. Recruitment Process

To recruit participants for capturing hand gestures in New Zealand Sign Language (NZSL), various methods were employed, including emails, phone calls, and in-person presentations. Physical presentations were conducted at different educational centers to inform potential participants about the study’s objectives. Along with these presentations, research approval letters from the administration and detailed privacy guidelines were shared to ensure transparency and address any concerns related to the study. This helped build trust and promote participation among the NZSL community.

Despite these efforts, several challenges arose during the recruitment phase. Some centers were overwhelmed with their workloads and were unable to commit to the project. Additionally, certain potential participants expressed concerns about recording their hand gestures on video, which made them reluctant to participate. Addressing these concerns and workload constraints required patience and ongoing communication.

After four weeks of persistent outreach, 14 participants agreed to take part in the study and record their hand gestures. To respect their time and minimize any inconvenience, we ensured that each participant spent the least amount of time possible during the recording process. Breaks were provided between data capture sessions to ensure participants remained comfortable and engaged throughout the process. This approach allowed for an efficient yet considerate data collection experience.

### 3.5. Implementation Details and Code Availability

The system was developed using Python 3 and several open-source libraries, including MediaPipe for hand landmark detection, OpenCV for real-time image processing, and Scikit-learn for gesture classification. Model training was conducted using traditional machine learning classifiers.

The application was designed to run on cross-platform environments including Windows, Ubuntu, and macOS. The minimum recommended system configuration includes an Intel i5 or Ryzen 5 CPU, 8 GB RAM, and an integrated or external webcam.

To ensure reproducibility and to support further research, the complete source code, including documentation, sample gesture images, and dataset formats, is publicly available in the project’s GitHub (version 3.14.4) repository:

https://github.com/mubashir4ali/nzsl (accessed 11 October 2024)

The repository includes the following components:

- CaptureData.py–for collecting hand gesture data using webcam.
- DetectLetter.py–for real-time letter detection.
- Analysis.py–for comparing algorithm performance.
- requirements.txt–for installing all dependencies.
- Sample gesture images and a dummy dataset file in CSV format.

A detailed README.md is provided in the repository, outlining setup instructions, manual preprocessing steps, and future extension suggestions. Users are encouraged to collect their own gesture data following the reference format provided in the CSV sample. The dataset used in this study is not publicly released due to privacy concerns; however, the repository includes sample entries to facilitate replication.

## 4. Dataset Generation

We methodically planned and executed a number of features to enable smooth exchange of messages among sign language users and others who depend on spoken language in order to bring the Sign Language Translation system to life. Two primary modalities comprise the implementation: Learning, New Zealand Sign Language Hand Gestures into Alphabets and Numbers. This study employed a quantitative approach to analyze hand gesture data for New Zealand Sign Language (NZSL) recognition. Data collection is done from the several places mentioned in the Section 3.1. Preprocessing included the use of tools like OpenCV and Mediapipe to extract key hand landmarks and features, ensuring the dataset was clean, standardized, and suitable for analysis. The solution design involved exploring machine learning techniques to develop and evaluate models capable of accurately recognizing NZSL gestures in real-time. This study employs a structured research design to develop and evaluate a machine learning-based system for recognizing New Zealand Sign Language (NZSL) hand gestures. The research is divided into several key phases to ensure comprehensive development and assessment of the system. Initially, the project involves creating a dataset of approximately 100,000 hand gesture records, with each entry annotated by the calculated distances between key hand landmarks as well as touch-points of both hands and their distances. This extensive dataset serves as the foundation for training and testing various machine learning models. The primary focus is on evaluating models such as RF, NB, KNN, AB, SVM, DT, and LR to determine their efficacy in recognizing NZSL.

### 4.1. New Zealand Sign Language Alphabets to Text

Users have a choice of input options, including using their smartphone camera or webcam. Real-time video frame capture incorporated into the program, and MediaPipe’s Hand module utilised for accurate hand gesture identification. Instantaneous sign language alphabets and numbers to text translation has made possible by the application, which recognises sign language letters based on motions and displays them on the screen. For an improved user experience, a real-time video depiction of the hand motions is also be available.

**Figure 6:**
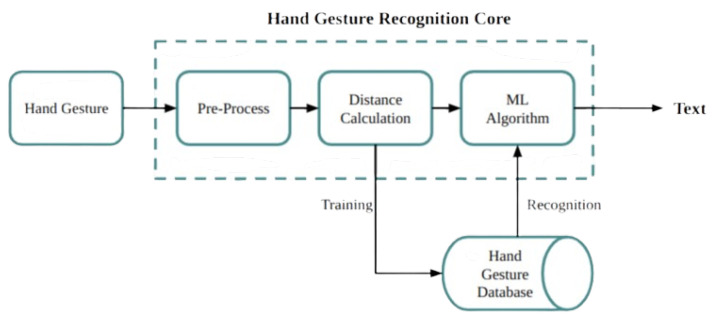
The Gestures in Sign Language.

The gestures in sign language that are recorded by a camera are analysed by a computer vision system. The recognition system identifies signs through analysis of hand and finger positions, shapes, and dynamic motion patterns. Machine learning algorithms are then read these recognised signs and convert them into written text, see Figure 6.

### 4.2. Research and Solution Design

This study employs a structured research design to develop and evaluate a machine learning-based system for recognizing New Zealand Sign Language (NZSL) hand gestures. The research is divided into several key phases to ensure comprehensive development and assessment of the system. Initially, the project involves creating a dataset of approximately 100,000 hand gesture records, with each entry annotated by the calculated distances between key hand landmarks as well as touch-points of both hands and their distances. This extensive dataset serves as the foundation for training and testing various machine learning models. The primary focus is on evaluating models such as Random Forest, Naive Bayes, k-Nearest Neighbor, AdaBoost, Support Vector Machine, Decision Tree, and Linear Regression to determine their efficacy in recognizing NZSL.

#### Solution Design

**Figure 7:**
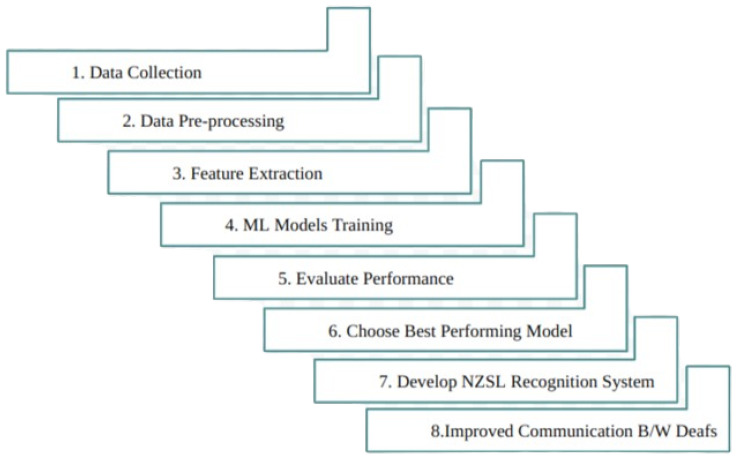
Workflow for Developing the NZSL Recognition System.

Figure 7 explains the workflow for developing the NZSL recognition system involves: data collection, pre-processing, feature extraction, ML model training, performance evaluation, selecting the best model, and building the system to enhance communication for the deaf.

- Programming Languages: Python for developing machine learning models and application integration.
- TensorFlow, Scikit-learn, and Keras for model training and evaluation.
- OpenCV and MediaPipe for hand gesture detection and analysis.
- CSV files for dataset storage and management.
- Tools for developing a mobile or web-based application that integrates real-time gesture recognition.

### 4.3. Explanation of Dataset and Procedure of Data Collection

The dataset used in this study includes hand signal data in the context of New Zealand Sign Language (NZSL). The data gathering process entails recording real-time hand motions using a camera interface. Participants are asked to execute a variety of NZSL gestures, which are then recorded and preserved for study. In NZSL, each gesture corresponds to a specific letter of the alphabet, allowing for the construction of a comprehensive recognition system.

**Figure 8:**
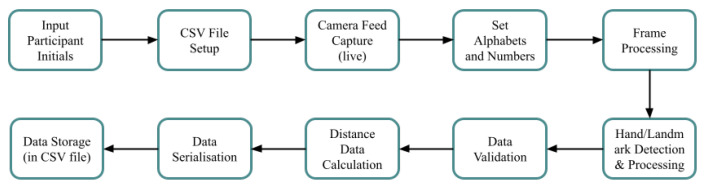
Image Data Capturing Process.

In the Figure 8, the process begins with participant input and file setup, followed by webcam feed capture. The MediaPipe framework is utilized to detect hands, extract precise landmark points, and process frame data at predefined intervals. The letter selection and frame processing branch ensures proper detection of hand gestures, calculating distance data and serializing it into a CSV format, including fields for tip points and other features. Finally, the data is securely stored for use in training machine learning models. Each phase is color-coded for clarity.

Then the output of those is saved into the CSV file, where the CSV header have 59 columns. The first column is the letter (e.g., A–Z), and the others are (unit-0 to unit-55), where unit-0 to unit 27 covers distances of hand 1 and unit-28 to unit-55 for hand 2, which covered the distances between different landmarks. Table 2 provides an explanation of what each value signifies in the unit-* columns. In addition, storing the fingertips information because of the similar hand landmarks for Vowels A, E, I, O, U.

**Figure 9:**
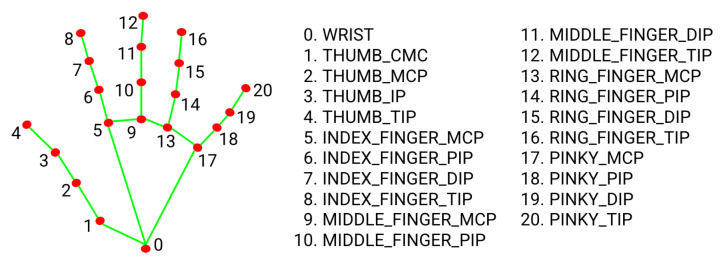
Hand Landmarks.

These distances are measured relative to a standard reference length, typically the distance between the wrist and the base of the thumb. They provide insights into the shape and configuration of the hand, which can be utilized for various applications such as hand gesture recognition and sign language interpretation, also demonstrated in Figure 9.

#### 4.3.1. Dataset Diversity and Recording Conditions

The dataset was constructed to ensure a high level of diversity in gesture representation. It includes contributions from fourteen participants (93% female, 7% male) aged between 40 and 60 years, who performed gestures under a variety of conditions:

- **Position**–Gestures were recorded at different angles (frontal, lateral) and distances (30 cm to 1 m from the camera).
- **Lighting/Backgrounds**–Multiple environments (e.g., bright/dim lighting, cluttered/plain backgrounds) were used to enhance robustness.
- **Hand Dominance**–While the initial dataset focused on right-handed gestures, future work will include left-handed variants to improve inclusivity.

This variability ensures the model generalizes well to real-world scenarios.

#### 4.3.2. Relevance

The dataset’s relevance lies in its alignment with the research objective of automatically recognizing NZSL alphabets using machine learning techniques. By capturing real-time hand movements and extracting landmark distances, the dataset facilitates the training and evaluation of machine learning models for NZSL recognition. Its focus on NZSL gestures ensures that the data accurately reflects the nuances and intricacies of sign language communication, making it highly relevant to the research context.

#### 4.3.3. Explanation of Landmark Detection and Feature Extraction Using OpenCV and Mediapipe in Python

**Figure 10:**
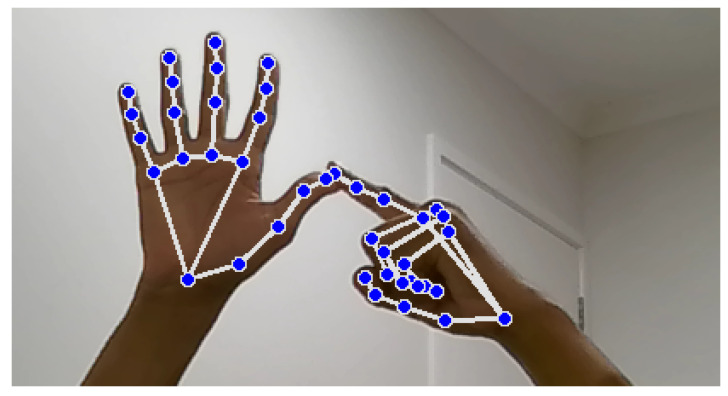
New Zealand Sign Language–Letter A.

**Figure 11:**
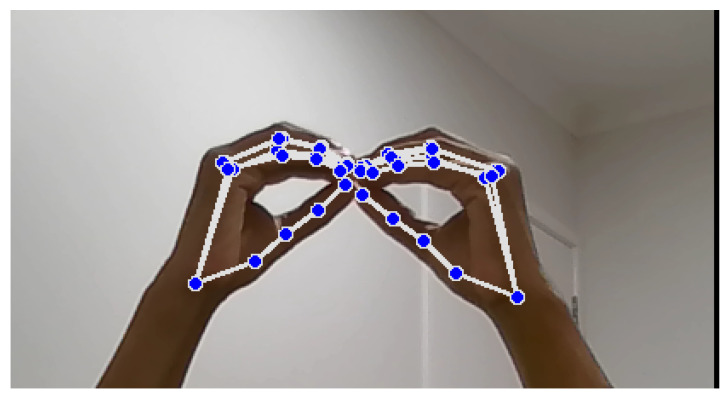
New Zealand Sign Language–Letter B.

Landmark identification and feature extraction, as illustrated in Figure 10 and Figure 11, are crucial steps in the hand signal recognition process. These figures, captured using images of my own hands, demonstrate the steps involved in gesture recognition. This is achieved using the Python packages OpenCV and MediaPipe. The MediaPipe library provides pre-trained models for hand landmark identification, enabling the extraction of key points that represent the hand’s spatial configuration in real time during gesture execution. These landmarks serve as features for further analysis and classification. The resulting dataset is then utilized to train machine learning models for NZSL recognition.

During dataset collection, images were captured in various indoor environments with natural and artificial lighting to simulate real-world variability. While Figure 10 illustrates typical hand positioning, not all participants had identical hand orientations or background settings. Some recordings included background noise such as household items or furniture. To enhance model robustness, the dataset intentionally includes gestures taken at different angles, lighting intensities, and backgrounds, introducing natural visual noise. Participants were instructed to maintain clear hand visibility, but minor occlusions or distractions were not filtered out to reflect practical usage scenarios.

The Mediapipe hand tracking library uses a deep learning-based approach to track and detect human hands in real time. It is powered by a lightweight convolutional neural network (CNN) model trained on millions of labeled hand images to recognize hand landmarks and their relationships. The network is optimized for efficiency and can run on both desktop and mobile devices.

(1) $$\mathrm{distance}=\sqrt{{\left(p_{1x}-p_{2x}\right)}^{2}+{\left(p_{1y}-p_{2y}\right)}^{2}}$$

Equation (1) computes the Euclidean distance between two points, denoted as $p_{1}$ and $p_{2}$ , in a two-dimensional plane. The x-coordinate of $p_{1}$ is represented by $p_{1}x$ , while its y-coordinate is p1y. Similarly, $p_{2}x$ and $p_{2}y$ correspond to the x and y coordinates of $p_{2}$ , respectively.

(2) $$\mathrm{relative}\;\mathrm{distance}=\frac{\mathrm{distance}\;\mathrm{between}\;p_{1}\;\mathrm{and}\;p_{2}}{\mathrm{standard}\;\mathrm{length}}$$

Equation (2) determines the relative distance between two points, $p_{1}$ and $p_{2}$ , in relation to a given standard length. This calculation is achieved by dividing the Euclidean distance between $p_{1}$ and $p_{2}$ by the specified standard length.

To check if the calculated distance is less than a given threshold TOUCH_THRESHOLD_PIXELS, which is a constant value (30 pixels here). Mathematically, this is represented as:

If distance Less Than TOUCH_THRESHOLD_PIXELS then fingertips are considered to be touching.

(3) TOUCH_THRESHOLD_PIXELS=30

To calculates the Euclidean distance between two points on an image plane to determine if fingertips from each hand are close enough to be considered touching. For two points point1 and point2, the formula is (4):

(4) $$\mathrm{TP}\;\mathrm{dist}=\sqrt{{\left(\left(x_{1}\times im\_W-x_{2}\times im\_W\right)\right)}^{2}+{\left(\left(y_{1}\times im\_H-y_{2}\times im\_H\right)\right)}^{2}}$$

where:

- ( $x_{1}$ , $y_{1}$ ) are normalised coordinates of point1.
- ( $x_{2}$ , $y_{2}$ ) are normalised coordinates of point2.
- im_W and im_H are the dimensions of the image, scaling the normalised values to pixel values.

Once the hand landmarks are discovered, the library can use them for gesture detection, hand posture estimation, and augmented reality. The library also provides a variety of tools for processing and visualising the hand landmarks, such as the capacity to compute hand attributes, eliminate noisy landmarks, and annotate hands. We used a function that stated as HandImageToDistanceData of the input image I into a feature vector D to compute the distances between various landmarks on the detected hand(s):

(5) D=HandImageToDistanceData(I)

#### 4.3.4. Data Preprocessing

The package used in this case is called Pandas. During the landmark record, when no hand expression was shown on the recording screen, there were empty values in the entire row while data was being captured against each alphabet. Since those rows were making no contributions, they were eliminated completely. Once the data was captured, duplicate records and outliers were also eliminated [39].

Data normalisation is an important preprocessing step in machine learning and data analysis. It involves transforming the data to fall within a certain range, typically [0, 1], to ensure that each feature contributes equally to the analysis and modelling. Normalising can help improve performance.

(6) $$X_{ij,normalized}=\frac{x_{ij}-x_{j,min}}{x_{j,max}-x_{j,min}}\;$$

In Equation (6) is the original value. Minimum value of the column j is $x_{j}min$ and the maximum value of the column j is $x_{j}max$ . Numerator shifts the data in column j so that the minimum value becomes 0. Denominator does the subtraction on the column j ranges. Dividing the shifted values by the range scales the values to fall within the range [0, 1].

## 5. Application

Gesture recognition systems are increasingly utilized in crisis management and disaster relief contexts, where they enhance interpersonal communication and support collaborative decision-making [40]. These systems allow users to convey information intuitively, minimizing the need for specialized terminology [41]. Additionally, the automotive industry is exploring and implementing advanced AI systems by using image acquisition technology to detect gestures, processing them against a predefined set of gestures, and then sending commands based on the detected gestures. However, this represents just the initial stage, as only a limited number of functions have been introduced to the market; ongoing research and development are essential [42]. Another prominent area of application is Human-Robot Interaction (HRI), where gesture recognition plays a critical role. The integration of hand gestures with voice commands enhances precision in detecting user intent, supports seamless navigation, and facilitates the execution of routine tasks. Beyond usability, gesture recognition contributes to improved data validation and system reliability. These advancements are expected to encourage broader investment across sectors seeking intelligent, intuitive interaction frameworks [41].

### 5.1. Challenges and How They Were Addressed

A key challenge encountered during the development of the NZSL gesture recognition system was participant hesitancy in contributing to the dataset. Privacy concerns, particularly related to recording hand gestures, were frequently cited. Time constraints and scheduling conflicts further discouraged participation. This limitation significantly affected the data acquisition phase, given that the system’s accuracy and robustness depend on a sufficiently large and diverse dataset to train and validate the machine learning models. To address these concerns, I implemented several strategies to make the data collection process more participant-friendly and alleviate their worries. To ease privacy concerns, detailed demonstrations were provided to participants, showing how the recorded hand gesture data would be used. These demos illustrated the process from data collection to its conversion into anonymized landmark points, which reassured participants that their personal identity would not be associated with the gesture data. Furthermore, the data collection process was kept as short and efficient as possible, reducing the amount of time participants needed to contribute. By streamlining the data capture to focus on key gestures and reducing redundancies, the research team managed to collect sufficient data without causing significant inconvenience to participants.

#### 5.1.1. System Development

**Figure 12:**
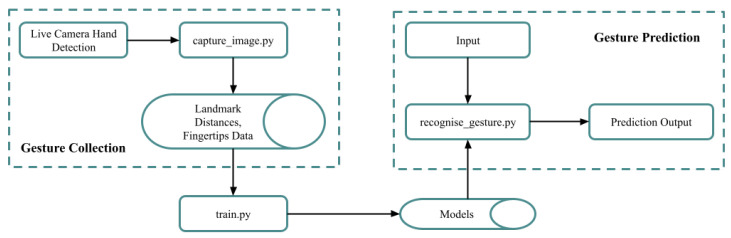
Application Block Diagram.

The Figure 12 depicts a block diagram of a gesture recognition application. The process begins with a live camera capturing hand images. These images are then processed to detect hand landmarks, calculate distances between them, and extract data from fingertips. This information is used as input for a gesture prediction model, which predicts the performed gesture. The model is trained using a separate “train.py” script, which presumably utilizes a dataset of gestures and their corresponding labels to learn the patterns and relationships between hand features and gestures. The overall diagram illustrates the flow of data and the key components involved in recognizing gestures from real-time video input.

#### 5.1.2. Collection of Data and Captured Dataset

This research aims to translate a dual-handed sign language, New Zealand Sign Language (NZSL). While there are available sign language gesture datasets for single-handed sign languages, such as ASL and PSL, comprehensive gesture datasets for dual-handed sign languages are lacking. To train the machine learning system effectively for translating the hand signs of NZSL, a high-quality classification dataset is essential. However, the literature review revealed no existing database relevant to NZSL image sets. As a result, the necessary classification dataset has been collected to facilitate training.

**Figure 13:**
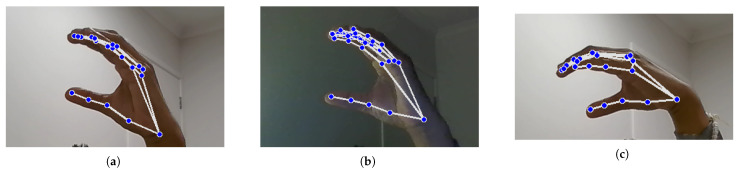
Example illustrating the variations in lighting conditions during the capture of the gesture for the letter “C.” (Extracted from the training dataset of the proposed New Zealand Sign Language translation system). (**a**) Bright Background. (**b**) Dark Background. (**c**) Different Angle.

In order for the trained system to operate at its best, intra-class differences in the form of shape, rotation, and orientation must be present in the input dataset [43]. Within the same data class, signers in this dataset rotated gestures and changed hand angles. Additionally, altering the background and lighting during the sign gesture recording process can improve the overall accuracy of the sign language translator [44]. Therefore, a variety of lighting settings, background modifications, sign rotation, background obstacle addition, and sign shape modification were used to gather the dataset for this proposed New Zealand Sign Language translator. Some instances of the intra-class changes found in the dataset are shown in Figure 13, which feature images of my own hands.

#### 5.1.3. Pre-Processing and Training of Data

In developing a sign language recognition system using MediaPipe and OpenCV, data collection is a critical first step. The dataset comprises hand gesture data captured in real-time for both hands. Each gesture represents a specific letter in New Zealand Sign Language (NZSL). For the letter “C,” data is exclusively collected from one hand; however, for all other letters, gestures are captured using both hands to provide a comprehensive representation of the signs. The landmark distances for hand one are stored in columns unit-0 to unit-27, while those for hand two are recorded in unit-28 to unit-55. Additionally, the tooltip distances, which represent the spatial relations of key points, are also saved for further analysis.

Data cleaning is essential to ensure the reliability and accuracy of the sign language recognition model. This process begins with the removal of empty data entries. Empty data points can occur due to missed frames or interruptions during the capturing process and may negatively affect model performance. Next, duplicate data entries are identified and eliminated to prevent skewing the training process. Duplicate data can arise from repeated gestures during the capture session, leading to overfitting the model. Irrelevant fields were removed from the dataset, retaining the most significant features while discarding less relevant data. To increase the robustness of the model, techniques like rotation of data for hand 1 and hand 2 used to cater left hand and right hand combinations.

Outliers are another concern when cleaning the dataset. Outliers can occur due to anomalies in the data collection process, such as unexpected movements or camera misalignment. To identify and remove outliers, statistical methods can be employed, such as calculating the Z-score or using the Interquartile Range (IQR) method. The Z-score can be calculated as follows:

(7) $$Z=\frac{X-\mu }{\sigma }$$

where:

- Z is the Z-score
- X is the value being standardized
-
μ is the mean of the dataset
-
σ is the standard deviation of the dataset

Data points with a Z-score greater than 3 or less than −3 are often considered outliers and can be removed from the dataset.

#### 5.1.4. Algorithm Selection and Model Training

The selection of algorithms for the project focused on balancing accuracy, computational efficiency, and ease of implementation. Various machine learning algorithms were considered and evaluated for their ability to recognize New Zealand Sign Language (NZSL) gestures effectively. These included traditional methods such as k-Nearest Neighbours (KNN), Support Vector Machine (SVM), Decision Trees (DT), Naïve Bayes (NB), Random Forest (RF), AdaBoost (AB), Logistic Regression (LR), and Multi-Layer Perceptron (MLP). Each of these algorithms was chosen for its unique strengths in classification tasks, particularly in handling multi-class problems like gesture recognition.

The dataset, consisting of approximately 100,000 hand gesture samples, was crucial for training and testing the models. Each gesture was represented by hand landmark data, extracted using computer vision techniques. The dataset was split into training and testing subsets to ensure a fair evaluation of each algorithm’s performance. The features extracted from the hand landmark, such as distances between fingertips and other key hand points—were fed into the algorithms to classify the gestures.

Once the models were selected, they were trained using a Python script (“train.py”), which processed the dataset and extracted relevant hand features. Each algorithm was evaluated on multiple metrics, including accuracy, precision, recall, and F1-score, to determine its suitability for real-time hand gesture recognition. Model hyperparameters were tuned through experimentation, adjusting factors such as the number of decision trees in RF, the number of neighbors in KNN, and the learning rate for AdaBoost, to achieve optimal performance.

This comprehensive approach to algorithm selection and model training ensured the development of a robust and efficient system capable of recognizing NZSL gestures with high accuracy. Further modifications to the model architectures and parameters were explored to improve performance, with the goal of enhancing communication between the hearing and deaf communities.

### 5.2. Analysis of Solution Evaluation and Testing

The evaluation and testing phase of the NZSL gesture recognition system was a critical aspect of validating the effectiveness and accuracy of the chosen machine learning models. To ensure comprehensive evaluation, the system underwent several rounds of testing using both pre-recorded and real-time hand gesture data. Initially, a test set comprising 20% of the dataset, which included approximately 20,000 hand gesture samples, was separated from the training data to assess the generalization capabilities of the models. The performance of models such as Random Forest (RF), Support Vector Machine (SVM), k-Nearest Neighbours (KNN), and AdaBoost (AB) was measured using metrics like accuracy, precision, recall, and F1-score. This allowed for a balanced assessment of how well the system classified hand gestures, accounting for both correct and incorrect predictions…

Real-time evaluation was essential, given the system’s ability to analyze continuous video feeds for gesture detection from a camera feed and translate hand gestures into text in real-time. This aspect of the testing focused on evaluating the system’s latency, responsiveness, and prediction accuracy in dynamic environments. During these tests, participants from the intervention group were asked to perform various NZSL gestures, while the system predicted the corresponding letters or words. A post-test analysis was conducted to compare the recognition accuracy and user proficiency improvements after system usage, with accuracy rates exceeding 90% in controlled environments. These real-time tests confirmed the system’s ability to effectively bridge communication gaps, with the added benefit of immediate feedback for the user to improve their gesture performance. Through continuous iterations and parameter tuning, the system achieved enhanced accuracy, robustness, and a seamless user experience.

### 5.3. Challenges and How They Were Addressed

One of the significant challenges faced during the development of the NZSL gesture recognition system was participant reluctance to contribute to the dataset. Many participants expressed concerns about privacy, especially regarding the recording of their hand gestures. Additionally, some were hesitant to commit time to the data collection process due to their busy schedules. This posed a serious limitation as the system relied heavily on a large, diverse dataset to train the machine learning models effectively. To address these concerns, I implemented several strategies to make the data collection process more participant-friendly and alleviate their worries.

To ease privacy concerns, detailed demonstrations were provided to participants, showing how the recorded hand gesture data would be used. These demos illustrated the process from data collection to its conversion into anonymized landmark points, which reassured participants that their personal identity would not be associated with the gesture data. In addition, the data collection protocol was intentionally optimized for brevity and efficiency, ensuring minimal time commitment from each participant. The capture process prioritized essential gesture recordings and eliminated procedural redundancies, allowing the research team to acquire a representative dataset without imposing undue burden on contributors.

Another challenge was the system’s initial difficulty in recognizing complex hand gestures in real-time scenarios. Early versions of the system struggled with gestures involving both hands or those that were performed at varying angles. To overcome this, the machine learning models were retrained with a more diverse set of hand gesture data, including multiple angles and lighting conditions. Additionally, modifications to the feature extraction process, such as fine-tuning the hand landmark detection algorithms using OpenCV and MediaPipe, improved the system’s robustness in detecting subtle hand movements. This allowed for more accurate recognition of gestures, even in non-ideal conditions, significantly enhancing the system’s performance during live tests.

### 5.4. Ethical Considerations

Ethical considerations are paramount when conducting research involving sign language data, as it directly impacts the privacy and well-being of the signing community. Ensuring the confidentiality and privacy of individuals and organizations whose data is analyzed is crucial. The study adheres to ethical standards by securing legitimate and authorized data sources and using a balanced dataset to represent diverse demographics within the signing community, thereby minimizing bias. Fairness measures were implemented during model evaluation to identify and address any algorithmic bias.

Personal identifiers were removed during data collection to maintain anonymity. Access to New Zealand Sign Language (NZSL) data is restricted to authorized researchers, with detailed audit logs kept to monitor access and usage. Only the researcher, had access to the consent forms, which are stored securely in a locked cabinet and digitally on encrypted Microsoft Azure Storage, accessible only to the researcher. When no longer needed, consent forms are securely destroyed, either shredded or digitally deleted. The research complied with the ethical guidelines established by the National Bioethics Committee of New Zealand. Intellectual property rights, data sovereignty, and indigenous knowledge related to NZSL have be respected. Informed consent have been obtained from participants, with data anonymized to protect privacy by recording only numerical values representing landmark distances from hand gestures. Efforts were be made to ensure a diverse participant pool to avoid dataset bias.

All hand gesture data has been anonymized, removing personal identifiers before analysis. The data has been securely stored on encrypted hard disks and Microsoft Azure Storage, with encryption, access controls, and regular backups to ensure high levels of data protection and accessibility. Only authorized personnel had access to the data, and strict controls are in place to prevent unauthorized access. Aggregated and anonymized data might shared or published, but no identifiable information have be given to third parties. The data is used solely for the research project’s objectives: developing and evaluating a application for recognizing NZSL Alphabets. It is not be shared with external parties or used for commercial purposes. By following these procedures, the researcher aims to safeguard the integrity and confidentiality of the collected data.

#### Ethical Approvals and Conditions

Before commencing the research project, ethical approval was obtained from the Research & Ethics Committee at Whitecliffe. The proposal included detailed information on participant recruitment, informed consent, and privacy safeguards. Teachers and trained individuals in NZSL were recruited through emails, phone calls, and presentations. Ethical considerations involved ensuring participants’ anonymity by only recording hand gesture landmarks and not collecting personally identifiable information. The consent process was clearly outlined, with both verbal and written consent obtained before data collection. Furthermore, the project follows strict data security protocols, storing all collected data on encrypted Microsoft Azure Storage and only allowing access to authorized personnel. After completing the study, all data have be retained securely until 2029 for further analysis or publication, after which it will be permanently destroyed to ensure data privacy.

## 6. Results and Analysis

Following the introduction of the system’s architecture and working principles, it is essential to demonstrate the research’s progress and effectiveness in achieving its objectives related to New Zealand Sign Language (NZSL) recognition. To accomplish this, the first step involved showcasing the system’s real-time performance in NZSL detection to highlight the study’s outcomes. Subsequently, the research goals have be addressed by presenting the key performance indicators for the implemented NZSL recognition system.

### 6.1. Design and Evaluation Measures

**Figure 14:**
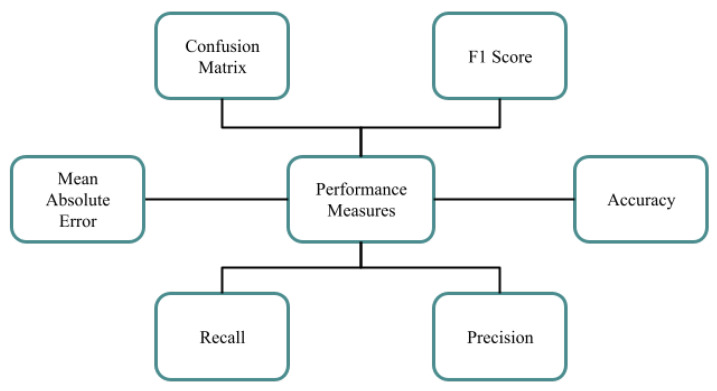
Important Performance Measures.

Important performance measures play a crucial role in guaranteeing system efficiency by acting as a link between strategic goals and accomplished results. The performance measures that have been identified for the suggested New Zealand Sign Language translation system are covered in this section. For NZSL project, evaluating the machine learning algorithm is essential. Even though the accuracy of the created model has demonstrated promising outcomes, when other metrics are taken into account, relying exclusively on accuracy scores may lead to false conclusions. Classification accuracy is frequently used as the main performance metric for models, although it is not enough for a thorough analysis on its own. Therefore, the best method for determining the model’s efficacy is to evaluate a set of performance metrics. The map of performance indicators, such as the confusion matrix, F1 score, accuracy, and Mean Absolute Error, Precision, used to assess the suggested New Zealand Sign Language translator is shown in Figure 14.

#### 6.1.1. Confusion Matrix

The confusion matrix is a crucial tool for evaluating the performance of a classification model. It provides a detailed breakdown of the model’s predictions compared to actual outcomes, allowing us to see not only the overall accuracy but also where the model may be making specific types of errors. The matrix displays the distribution of predictions across four categories: True Positives (TP), True Negatives (TN), False Positives (FP), and False Negatives (FN). Each of these elements provides insight into the model’s classification behavior, which is especially useful in applications where specific types of errors have distinct consequences [45].

- **True Positive (TP)**: These are cases where the model correctly predicted the positive class. For example, if a model is designed to detect fraud, a true positive would mean that an actual fraudulent transaction was correctly identified as fraud.
- **True Negative (TN)**: True negatives occur when the model correctly predicts the negative class. In the case of fraud detection, this would mean that a legitimate transaction was accurately classified as non-fraudulent.
- **False Positive (FP)**: Also known as a Type I error, a false positive happens when the model incorrectly predicts the positive class. Using the fraud example, this would mean a legitimate transaction was incorrectly classified as fraudulent. False positives can lead to issues such as unnecessary alarms or wrongful classifications.
- **False Negative (FN)**: Known as a Type II error, a false negative occurs when the model incorrectly predicts the negative class. In fraud detection, this would mean a fraudulent transaction was classified as legitimate, potentially allowing fraud to go undetected.

A confusion matrix is typically displayed in a table format, where rows represent the actual values (True/False) and columns represent the predicted values (True/False). This matrix format makes it easy to see where the model succeeded and where it failed, which can guide further tuning and optimization of the model. Table 3 shows onfusion Matrix for Actual and Predicted Values.

Where:

- **TP**: represents correctly identified positive instances.
- **TN**: represents correctly identified negative instances.
- **FP**: represents incorrect predictions where the actual class is negative, but the model predicted positive.
- **FN**: represents incorrect predictions where the actual class is positive, but the model predicted negative.

#### 6.1.2. Accuracy

Accuracy is a fundamental metric for evaluating the performance of a classification model. It represents the proportion of correctly classified instances out of the total instances and serves as a straightforward indicator of model effectiveness. Calculated as the ratio of true positives and true negatives to the total number of predictions, accuracy is particularly useful when classes are balanced.

(8) $$\mathrm{Accuracy}=\frac{Tp+Tn}{Tp+Tn+Fp+Fn}$$

#### 6.1.3. Precision

Precision measures a model’s accuracy in predicting positive instances by assessing the ratio of true positives to the sum of true positives and false positives. It reflects how many of the instances classified as positive are actually correct, making it essential in scenarios where false positives are costly, such as medical diagnostics or fraud detection. Precision helps ensure that positive predictions are reliable, though it’s often used alongside recall to get a balanced view of a model’s predictive capability, especially in imbalanced datasets.

(9) $$\mathrm{Precision}=\frac{Tp}{Tp+Fp}$$

#### 6.1.4. Recall

Recall quantifies a model’s ability to correctly identify all relevant positive cases, measuring the ratio of true positives to the sum of true positives and false negatives. It is particularly useful in applications where identifying every positive instance is critical, as it shows how many actual positives the model successfully captures. A high recall is essential when missing true positives carries a significant risk, although it’s often evaluated alongside precision to balance accuracy in identifying both true positives and true negatives.

(10) $$\mathrm{Recall}=\frac{Tp}{Tp+Fn}$$

#### 6.1.5. F1 Score

Evaluation metrics like recall, accuracy, precision, and F1 score are crucial for evaluating how well machine learning algorithms work. When working with unbalanced datasets, accuracy provides an overall measure of correctness, but it might not be enough. Recall emphasises the model’s capacity to catch positive instances, whereas precision concentrates on the accuracy of positive predictions [46].

The F1 score is a crucial metric that combines precision and recall into a single measure, providing a balanced evaluation of a model’s performance, especially in scenarios with imbalanced classes. It is calculated as the harmonic mean of precision and recall, offering a more comprehensive view of a model’s ability to correctly classify positive instances while minimizing false positives and false negatives. The F1 score is particularly valuable in applications where both false positives and false negatives carry significant consequences, ensuring that a model is not only accurate but also effective in identifying all relevant cases.

(11) $$F1=2\times \frac{\mathrm{Precision}\times \mathrm{Recall}}{\mathrm{Precision}+\mathrm{Recall}}$$

#### 6.1.6. Summary

To evaluate the research question, a real-time New Zealand Sign Language translator has been designed, built, assessed, and compared to the currently available sign language translators. This real-time translator is expected to recognize New Zealand human hand signs representing alphabets and numbers. The Design and Evaluation Measure identified in this chapter directly participate in the evaluation process of this real-time New Zealand Sign Language translator.

### 6.2. Presentation of Findings

In this section, we will evaluate the performance of various classification algorithms, including Random Forest (RF), Naive Bayes (NB), K-Nearest Neighbors (KNN), AdaBoost (AB), Support Vector Machine (SVM), Decision Trees (DT), and Logistic Regression (LR). Each algorithm will be assessed using key performance metrics: Score, Accuracy, Precision, and Recall, particularly in the context of recognizing New Zealand Sign Language (NZSL) alphabets.

The evaluation process will begin with a comprehensive analysis of the accuracy achieved by each algorithm. This will provide insights into their effectiveness in classifying the NZSL dataset. Following this, we will present the confusion matrices for each model, offering a detailed view of the correct and incorrect predictions made by the classifiers. The confusion matrix will serve as a valuable tool for understanding the models’ performance in distinguishing between different NZSL signs.

After identifying the best-performing model, we will discuss the results of pre-test and post-test evaluations conducted amongst participants using the application designed for NZSL recognition. These evaluations will be carried out through interviews, from which keywords will be extracted to assess participants’ understanding and proficiency in recognizing the target letters or symbols in NZSL. This comparison will highlight the impact of the model’s predictions on participants’ learning outcomes. The pre-test and post-test data will provide valuable insights into the effectiveness of the application and the learning improvements observed among participants.

**Figure 15:**
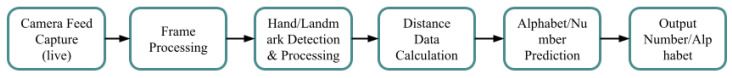
Image Data Distance Prediction Process.

Finally, we will delve into the performance of the best-performing algorithm, focusing on the scores for each NZSL alphabet. This detailed analysis will help in identifying the strengths and weaknesses of the chosen algorithm in recognizing and classifying each NZSL sign accurately. By the end of this section, we aim to provide a clear understanding of the comparative effectiveness of the evaluated algorithms, supported by quantitative metrics and visual representations, as well as insights into the practical application of the findings among the participants. Figure 15 shows image Data Distance Prediction Process.

#### 6.2.1. Rationale for Method Selection

While recent sign language recognition studies (Table 4) employ CNNs, RNNs, or Transformer-based approaches, these methods are typically feasible because the datasets are video-based and large-scale. In contrast, the NZSL dataset introduced in this study is relatively small and represented in a tabular format of extracted hand landmark distances. For such structured, low-dimensional data, classical machine learning methods are well-suited due to their efficiency, interpretability, and robustness in low-data regimes.

The specific set of classical models such as Random Forest, SVM, k-NN, Decision Trees, AdaBoost, and Naive Bayes were chosen to provide a comprehensive evaluation from multiple algorithmic perspectives. This diverse selection allows us to establish a robust and meaningful benchmark. For example:

- **Random Forest** and **AdaBoost**: ensemble baselines for tabular data.
- **SVM**: kernel-based, strong in complex spaces.
- **Decision Trees** and **Naive Bayes**: interpretable/lightweight.
- **k-NN**: instance-based benchmark.
- **Logistic Regression**: linear baseline.

By evaluating across these different paradigms, we can confidently assess the foundational performance of gesture recognition on our NZSL dataset. Importantly, applying deep architectures on the current dataset would likely result in overfitting and would not yield a fair or meaningful comparison with prior work. Once larger-scale raw video datasets of NZSL are available, we plan to extend this baseline with CNN- and RNN-based approaches for direct comparison with the state-of-the-art.

#### 6.2.2. Outcome Process

The feature vector D was passed to the top performing algorithm, i.e., Random Forest Classifier to get the predicted letter $\hat{y}$ .

(12) $$\hat{y}=RF\left(D\right)$$

In summary, the process can be mathematically represented as:

(13) $$\hat{y}=HandImageToDistanceData\left(I\right)$$

where:

- I is the input image
- HandImageToDistanceData is a function that converts the image I into a feature vector D.
- RF is the trained Random Forest classifier that predicts the letter based on D.

The results were really encouraging and can be useful in various ways.

- Real-time sign language alphabet detection: This system could be used to create applications for deaf and deaf people to interact more easily with physically normal people.
- Educational tools: This technology could be used to create interactive learning aids for teaching sign language.
- Accessibility enhancements: Integration with video conferencing platforms or the development of sign language captioning technologies may improve accessibility.

#### 6.2.3. Results of Experiments

Based on the results presented in Table 5: Various Machine Learning Results, a comparative analysis of the performance metrics—Accuracy, Precision, Recall, and F1 Score—was conducted for each classification model evaluated in this study.

From the data, the Random Forest Classifier emerges as the best-performing model, achieving an impressive Accuracy of 99.52%, along with corresponding metrics of Precision (99.53%), Recall (99.52%), and F1 Score (99.52%). This model’s performance indicates a strong ability to correctly classify the NZSL alphabets, making it a highly reliable choice for this recognition task. In contrast, other models exhibited significantly lower performance. The Support Vector Machine (SVM) recorded a mere Accuracy of 22.13%, coupled with a Precision of 19.28% and an F1 Score of 18.31%, indicating that it struggles to effectively distinguish between the classes in the dataset. Similarly, Logistic Regression and AdaBoost also underperformed, with accuracies of 28.59% and 7.19%, respectively. The results for these models suggest that they are not suitable for the current classification task involving NZSL alphabets. K-Nearest Neighbour (KNN) and Naïve Bayes showed moderate performance, with KNN achieving an Accuracy of 97.43% and Naïve Bayes scoring 96.96%. While these results are commendable, they still fall short of the Random Forest Classifier’s exceptional performance.

Given the evaluation results, the Random Forest Classifier is clearly the best choice for recognizing NZSL alphabets due to its high accuracy and balanced performance across all metrics. Its robustness in handling the complexities of the dataset, along with its ability to generalize well to unseen data, solidifies its selection as the optimal model for this application. Subsequently, this model will be further explored to analyze its performance in classifying individual NZSL signs and its overall effectiveness in enhancing the learning experience for users engaging with the NZSL recognition application.

#### 6.2.4. Accuracy of the Algorithms

**Figure 16:**
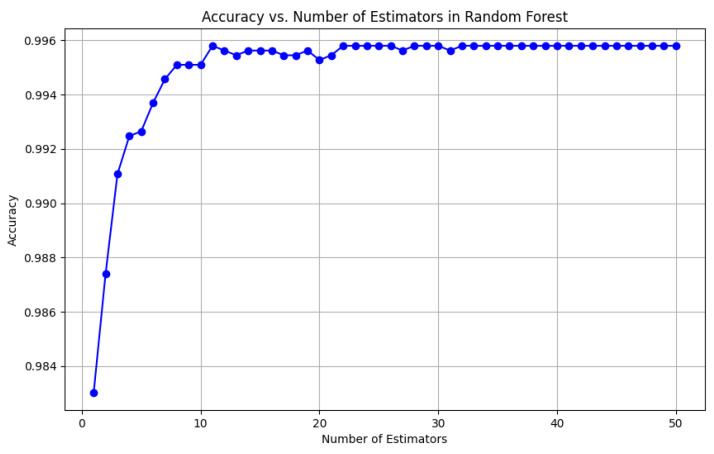
Random Forest Accuracy.

**Figure 17:**
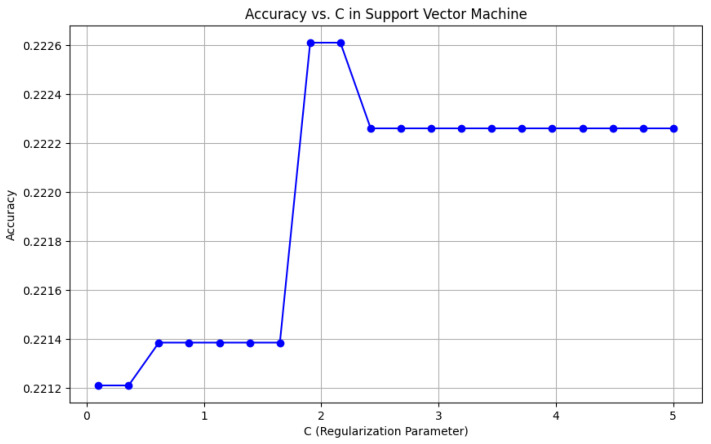
Support Vector Machine Accuracy.

**Figure 18:**
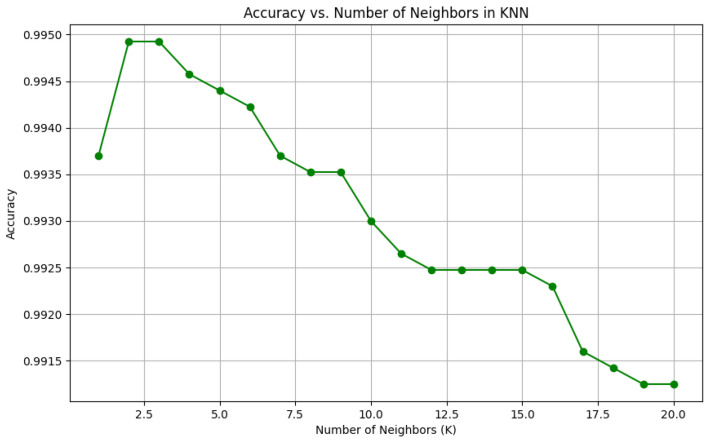
K-Nearest Neighbor Accuracy.

**Figure 19:**
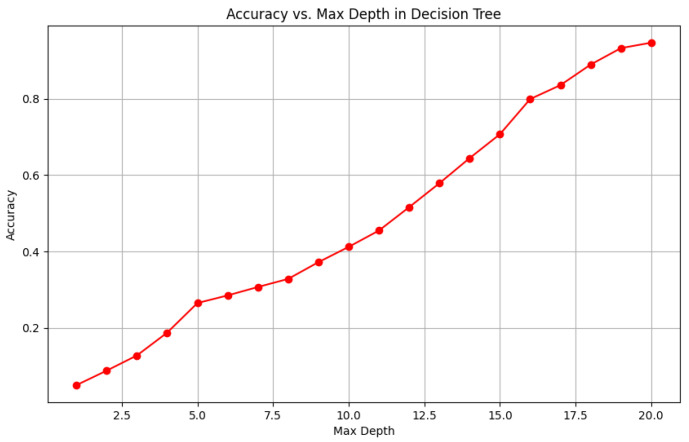
Decision Tree Accuracy.

**Figure 20:**
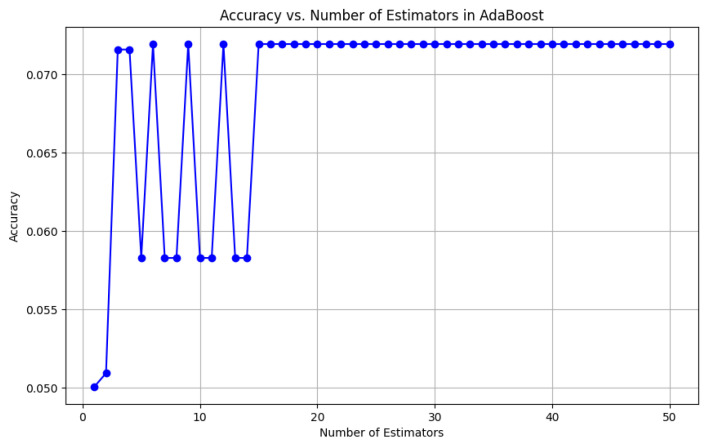
AdaBoost Accuracy.

**Figure 21:**
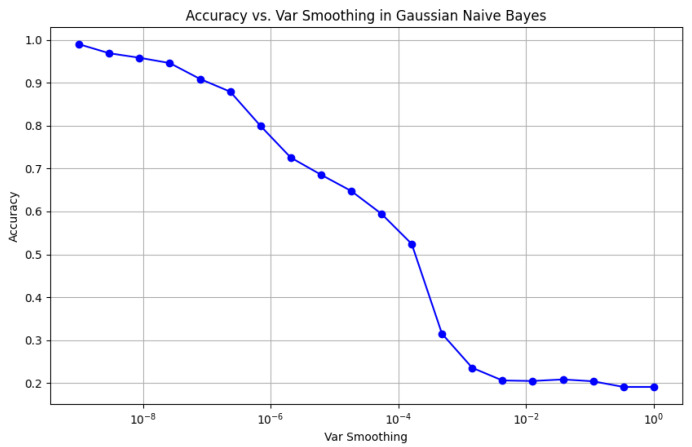
Naive Bayes Accuracy.

To visually represent the performance of the various classification algorithms, accuracy graphs are presented from Figure 16, Figure 17, Figure 18, Figure 19, Figure 20 and Figure 21, illustrating the accuracy achieved by each model in recognizing New Zealand Sign Language (NZSL) alphabets. These graphs provided a clear and concise comparison, allowing for an immediate assessment of how each algorithm performed relative to one another. By plotting the accuracy metrics alongside the corresponding algorithms, we can easily identify trends and patterns, highlighting the superior performance of the Random Forest Classifier compared to other models. This graphical representation enhanced the understanding of the results, making it easier to communicate the effectiveness of the chosen algorithms and support the conclusions drawn from the quantitative analysis.

#### 6.2.5. Confusion Matrix

**Figure 22:**
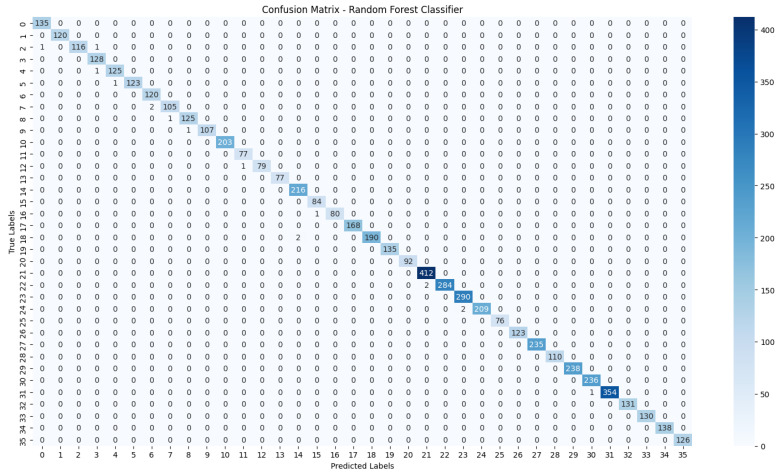
Confusion Matrix (Random Forest Classifier).

**Figure 23:**
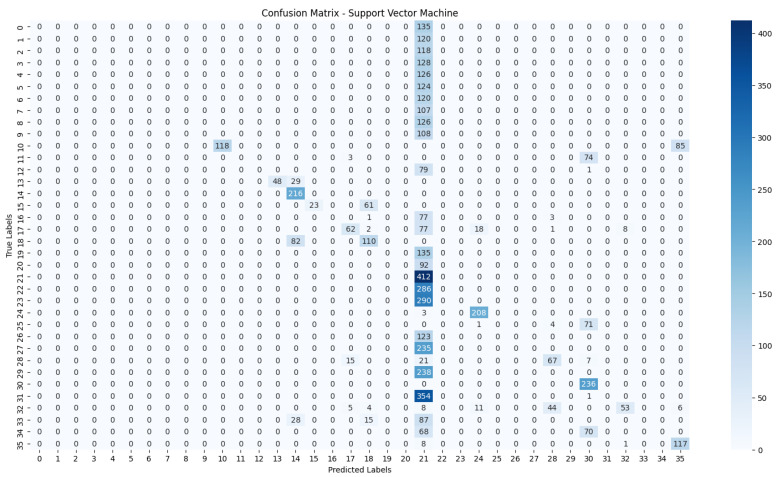
Confusion Matrix (Support Vector Machine).

**Figure 24:**
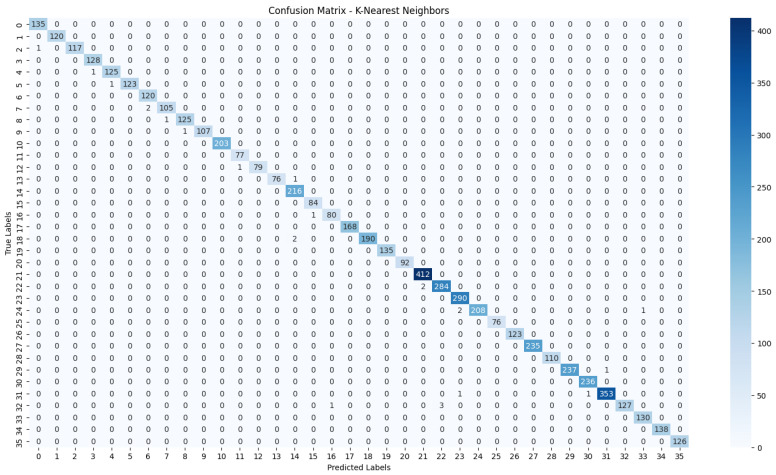
Confusion Matrix (K-Nearest Neighbor).

**Figure 25:**
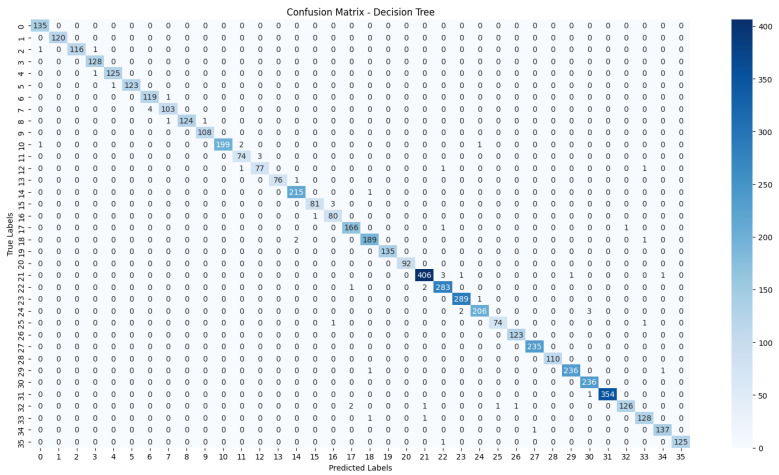
Confusion Matrix (Decision Tree).

**Figure 26:**
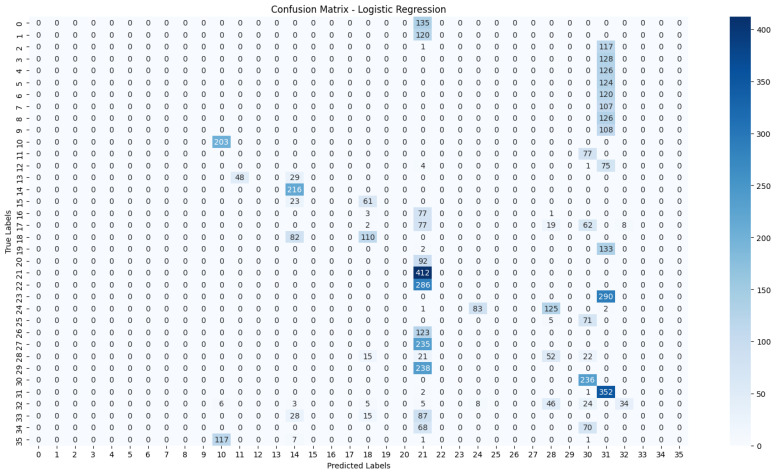
Confusion Matrix (Linear Regression).

**Figure 27:**
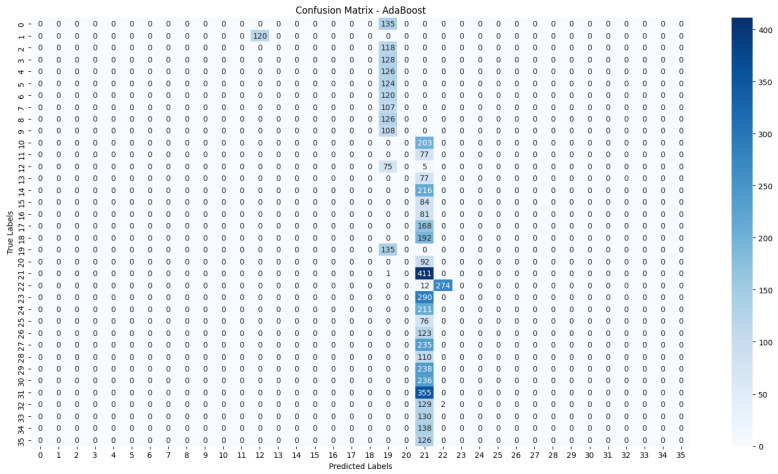
Confusion Matrix (AdaBoost).

**Figure 28:**
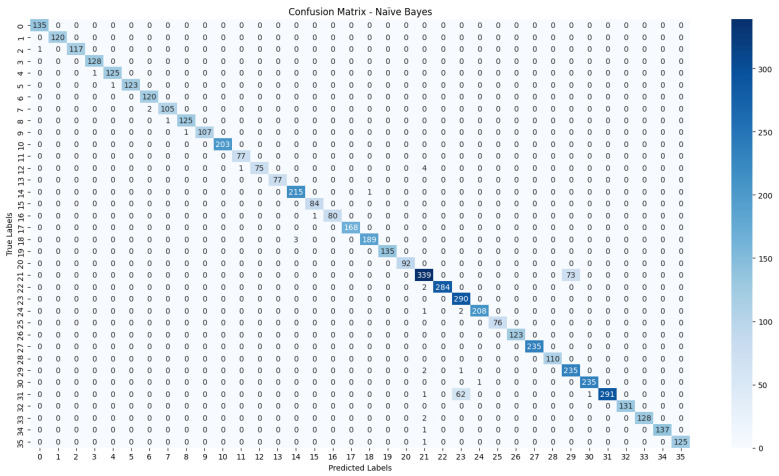
Confusion Matrix (Naive Bayes).

The confusion matrix is a pivotal tool in evaluating the performance of the various classification algorithms discussed, providing a comprehensive view of their predictive capabilities. For each model, the confusion matrix illustrates the number of true positive, true negative, false positive, and false negative predictions, allowing us to assess not only overall accuracy but also the model’s ability to differentiate between classes effectively. For instance, in the case of the Random Forest Classifier, the confusion matrix is expected to show a high number of true positives across the NZSL alphabet classes, reflecting its superior accuracy of 99.52%. In contrast, models like the Support Vector Machine (SVM) demonstrate a lack of class separation, evident in a higher count of misclassifications, as indicated by its low accuracy of 22.13%. By analyzing the confusion matrices, we gain insights into which classes are often confused with one another and where the models struggle, guiding future adjustments and improvements. This analysis not only highlights the strengths of the best-performing models but also reveals the weaknesses of less effective algorithms, emphasizing the importance of selecting a model that minimizes misclassifications and maximizes correct predictions in recognizing NZSL signs, as shown from Figure 22, Figure 23, Figure 24, Figure 25, Figure 26, Figure 27 and Figure 28.

Table 6 presents the F1-Scores for each alphabet (A–Z) and number (1–10) in the New Zealand Sign Language (NZSL) recognition system, showcasing the high accuracy of the model in classifying these characters, with most achieving perfect scores of 1.00.

#### 6.2.6. Hand Gesture Classification Using OpenCV and MediaPipe

Table 5 illustrates the superior performance of the Random Forest classifier, with excellent accuracy and precision, demonstrating its efficacy in identifying NZSL alphabets based on the provided attributes. Random Forest was followed by K-Nearest Neighbour, Naive Bayes and Decision Tree, suggesting that these algorithms are appropriate for the NZSL alphabet recognition test. AdaBoost did not perform as well as Support Vector Machine and Logistic Regression. This could be the result of a number of factors, including inadequate hyperparameter tuning or the algorithm’s intrinsic limits for this particular task.

As displayed in Figure 16, the proposed New Zealand Sign Language translator using a machine learning model achieved a final test accuracy of 99.52%. In machine learning, one of the key requirements is to prevent model overfitting. To determine if a model is overfitting, data scientists use a technique known as cross-validation, which involves splitting the data into two sections: a training dataset and a validation dataset. The training dataset is used to train the model, while the validation dataset is used to evaluate its performance. Metrics from the training dataset show the model’s progress during training, while metrics from the validation dataset measure the model’s output quality. Using this information, the overall test accuracy, training accuracy, validation accuracy, and cross-entropy at each step were identified for this study, as shown from Figure 16, Figure 17, Figure 18, Figure 19, Figure 20 and Figure 21.

## 7. Discussion

This aims to bridge the gap between research findings and the original research questions. By examining how the results align with the proposed hypotheses, we can gain a clearer understanding of the study’s significance and contributions to the field. Additionally, the chapter delves into the limitations and challenges encountered during the research process, offering valuable insights for future studies. Finally, we discuss recommendations to overcome these obstacles and enhance the robustness of future research endeavors.

New Zealand Sign Language gestures rely on dual-hand movements, making them more complex to interpret than single-handed sign languages. This research aims to test and validate the hypothesis that “Dual-handed sign languages, particularly New Zealand Sign Language, can be translated into text using human hand gestures.” Findings were achieved through an AI-based image processing system developed using machine learning techniques and implemented with OpenCV and MediaPipe. The system was programmed in Python.

After completing the retraining of the Random Forest model, a test accuracy of 99.1% was achieved, as shown in Figure 16. This test accuracy is expressed through the sensitivity and specificity of the trained system, supporting its reliability. A high test accuracy indicates that the system is well-suited to perform its designated task. This result reinforces the dependability of the findings, with the high test score essential for validating the research hypothesis.

In a real-time application, achieving a high classification score is essential to assess the system’s classification performance. Each letter or number received an individual classification score based on the metrics and algorithms applied. During the experimental phase of this research, classification scores were determined for each letter from A–Z and three additional numbers using gesture images as inputs. These scores were shown in Table 6. However, classification scores alone are insufficient to comprehensively measure model performance. Therefore, additional Key Performance Indicators have also been evaluated in this study.

The training and validation accuracy learning curves for this research, depicted in Table 5, indicate a well-fitting learning process of the trained system. This outcome reflects high accuracy values in the model’s overall performance as well as in the individual performance for each letter or number. These elevated accuracy values support the research hypothesis, demonstrating that gesture signs in dual-handed New Zealand Sign Language can be accurately translated into text format and displayed as complete sentences.

### 7.1. Need for a New Dataset

Most existing sign language datasets focus on ASL, ISL, or other single-handed systems, which are structurally different from New Zealand Sign Language (NZSL), a dual-handed language with unique gestures. As no public dataset adequately represents NZSL, we created a custom dataset to ensure cultural and linguistic relevance. However, due to ethical approval constraints and participant consent agreements, the dataset cannot be publicly released. A sample format and placeholder data are provided in the project repository to support reproducibility.

### 7.2. Critical Evaluation of Solution’s Novelty and Its Strengths and Limitations

Following are the limitations and challenges faced:

- Data Quality: There were be challenges related to data quality, such as noise, occlusions or variations in background conditions.
- Limited Sample Size: It is totally dependent on number of participants and gestures recorded.
- Bias and Representation: The dataset may have biases, notably in terms of the diversity and prevalence of NZSL gestures among groups or areas.

**Figure 29:**
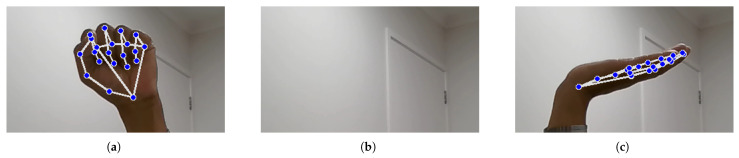
(**a**) Delete, (**b**) Nothing, (**c**) Space.

A limitation of this project is its inability to recognize words or phrases, as it only identifies NZSL alphabets or numbers. The dataset for this research study was assembled by executing a Python program named capture_image.py, designed to capture hand gestures using MediaPipe for landmark and fingertip detection. The program continuously tracks gestures with MediaPipe and OpenCV until the required number of gesture images is obtained, resulting in a dataset of approximately 100,000 hand gestures. However, gestures representing “Delete,” “Space,” and “Nothing” were not captured, which presents a limitation in the dataset, incorporating these gestures would improve model versatility and broaden its range of practical applications (see Figure 29).

While this study focused on isolated alphabet and number recognition, the framework can be extended to recognize words through sequential gesture classification. For example, words like “HELLO,” “THANK YOU,” or “YES” could be recognized by detecting and aligning a sequence of corresponding letter gestures or direct gesture signs (if data is available). Real-time applications could incorporate temporal segmentation and sliding window techniques to identify character sequences and map them to known words or commands. Implementing this would require labeled gesture sequences and an integrated language model to improve accuracy and contextual understanding.

A key limitation of this NZSL study is that it only captured right-handed gestures, excluding left-handed variations. This restriction may impact the accessibility and inclusivity of the NZSL application, as it does not fully accommodate left-handed users who may find it challenging to replicate gestures that are exclusively modeled for right-handed learners. Left-handed individuals often mirror gestures naturally, and without left-handed examples, they might face difficulties in achieving the same level of accuracy or comfort in learning NZSL. This limitation highlights the need for future versions of the application to include left-handed gesture recordings to ensure that the tool supports all learners equally, allowing for a more inclusive approach to NZSL education.

In the NZSL data capture process, a notable limitation is the gender distribution among participants: only 7% of participants are male, while the remaining 93% are female. This imbalance may affect the model’s ability to generalize effectively across different user demographics, as gender-related variations in hand shape, size, and movement patterns could introduce subtle biases. Consequently, the model’s performance might be optimized for characteristics more commonly found in female participants. Future work should aim to include a more balanced gender representation to improve the robustness and generalizability of the NZSL recognition system across diverse populations.

In NZSL, moving characters like the letters “H” and “J” present unique challenges due to their dynamic motion. These letters require precise movement that can be difficult for learners to replicate, especially in a digital or app-based environment where only static images or limited video guides are provided. Capturing the fluid motion of these letters is essential for accurate learning, as even slight deviations in the movement path or hand orientation can alter the meaning or clarity of the sign. Addressing this issue by incorporating slow-motion guides or interactive features to illustrate these movements could greatly enhance learners’ understanding and accuracy when practicing dynamic NZSL characters.

In developing the NZSL application with MediaPipe, the order of hand gestures was not initially monitored, which led to potential inconsistencies in recognizing left- and right-handed gestures. To improve accuracy, the system should consistently identify “hand1” as the left hand and “hand2” as the right hand when capturing gesture data. This distinction is essential for accurate gesture tracking, as it enables the system to differentiate between hands reliably, especially for gestures involving both hands or specific directional movements. By enforcing this hand identification protocol, the application can achieve a higher level of consistency and precision, making the learning experience more accurate and intuitive for users.

### 7.3. Critical Evaluation of Solution’s Impact on Legal, Cultural and Ethical Practices in the IT Industry

The integration of technology in teaching NZSL, particularly through the use of an application, has significant legal, cultural, and ethical implications in the IT industry. Legally, the application must comply with accessibility frameworks such as the New Zealand Disability Strategy and the Disability Discrimination Act. The app must comply with standards that make it usable for all learners, including those with disabilities. Additionally, data privacy laws, such as the Privacy Act 2020, must be considered when handling users’ personal data, especially in cases where the app collects gesture data or tracks user progress. Legal compliance is critical to protect both the users and the developers, ensuring that the technology remains a valuable tool without infringing on individuals’ rights.

Culturally, the NZSL app plays a vital role in preserving and promoting the New Zealand Deaf community’s language. The application helps bridge communication gaps, making NZSL more accessible to a wider audience, which can contribute to cultural preservation and respect for linguistic diversity. Ethically, the use of the app should be mindful of the diverse needs of users, ensuring that it serves not only as a learning tool but also as a means of fostering inclusivity. Developers must prioritize ethical considerations such as user consent, inclusivity, and non-discrimination when designing the app. Ensuring that the application accommodates both right- and left-handed users, and that it captures a wide range of gestures, is crucial to avoid marginalizing any group of learners. By considering these legal, cultural, and ethical factors, the NZSL app can positively impact the IT industry, promoting fairness, accessibility, and cultural respect.

### 7.4. Comparison with Previous Studies

Table 7 highlights the distinctions and similarities between the proposed NZSL recognition system and previously published studies. It emphasizes your focus on real-time, machine-learning-based NZSL gesture recognition, while other works focus on different languages, technologies, or types of gesture recognition.

### 7.5. Implications

The implications of integrating an NZSL application in education extend beyond just the enhancement of learning outcomes. Technologically, it promotes greater accessibility to New Zealand Sign Language, making it easier for both deaf and hearing individuals to learn and communicate. This could lead to a broader acceptance and understanding of the language, fostering a more inclusive society. However, the implications also include potential challenges, such as the need for continuous updates to accommodate evolving sign language norms or to address the limitations of current technology in accurately capturing subtle variations in hand gestures. Ethically, ensuring the app is designed with inclusivity in mind, considering factors like left- and right-handed variations, is crucial for providing a fair and equal learning experience for all users. Additionally, the legal implications surrounding data privacy must be carefully managed to protect users’ information and ensure compliance with relevant regulations.

#### Implications for Digital Health and Accessibility

One of the key implications of this research is its potential impact on digital health education and accessibility in healthcare settings. Effective communication between healthcare providers and patients is essential for accurate diagnosis, treatment, and patient engagement. Deaf individuals often face barriers in accessing healthcare services due to limited availability of NZSL interpreters. The development of AI-powered sign language recognition tools, such as the one proposed in this study, could serve as an assistive technology in hospitals, clinics, and telemedicine platforms.

## 8. Conclusions and Recommendations

This project report presents the development and outcomes of a real-time recognition system designed to address critical gaps in sign language technology, with a specific focus on New Zealand Sign Language (NZSL). Unlike most existing tools focused on single-handed alphabets, this system is tailored to handle NZSL’s dual-handed gestures. The project created a robust framework to enhance communication and accessibility for the Deaf community by collecting and processing a unique dataset of over 100,000 hand gesture landmarks. Using MediaPipe and OpenCV for landmark extraction and Random Forest as the classification algorithm, the model achieved high validation accuracy (up to 99.52%), demonstrating its effectiveness in recognizing complex, double-handed signs. The system’s resilience was verified by testing it under varying lighting conditions, rotated sign poses, and visual noise such as background obstacles. An emphasis was also placed on user experience and educational integration to support the wider NZSL learning community.

The evaluation confirmed that the system meets both technical performance metrics and real-world usability needs for NZSL users. Ethical considerations, such as data collection privacy and cultural sensitivity, were also addressed to ensure responsible implementation. This project ultimately delivers a technically sound, socially impactful solution, contributing to a more inclusive digital communication landscape for New Zealand’s Deaf population.

To train the model effectively, the project developed an original dataset since no public double-handed NZSL datasets existed. Data was collected under various lighting, gesture orientation, and occlusion scenarios to enhance robustness. A total of 100,000 hand landmark distances were generated using MediaPipe. The Random Forest classifier, selected for its balance between speed and accuracy, was employed to train the model. After generating bottleneck features, the model was trained and evaluated, with outputs reported through accuracy scores and cross-entropy loss values during each epoch, confirming strong generalization performance.

### 8.1. Contributions of the Research

This study makes several key contributions to the fields of machine learning, gesture recognition, and assistive communication technology. First, it introduces a curated dataset of 100,000 dual-handed NZSL gestures one of the largest and most diverse datasets of its kind. Second, it develops a functional real-time sign language recognition tool built with MediaPipe for feature extraction and classical machine learning models such as Random Forest, SVM, and KNN. Third, the research provides a working prototype of an educational application capable of translating signs into English text, with performance tested across various real-world conditions. In addressing practical challenges such as dynamic characters and symmetrical hand positioning, this work sets a foundation for future NZSL recognition tools and interactive learning applications.

### 8.2. Recommendations

To enhance the NZSL learning application, several improvements are recommended. First, it is essential to support both right and left handed gesture variations to accommodate a wider range of users. The dataset should be expanded to include high-utility gestures like “Delete”, “Space”, and “Nothing”, which are crucial for constructing complete sentences and practical communication. Dynamic gestures such as “H” and “J”, which involve motion rather than static hand shapes, should be addressed using slow-motion gesture capture or interactive guidance to improve recognition. Additionally, the application should integrate compliance checks with legal and ethical frameworks like the Privacy Act 2020, while embedding cultural sensitivity to enhance user trust and social acceptance.

### 8.3. Future Work

Future development of the NZSL recognition system should prioritize improving recognition of dynamic gestures through temporal modeling approaches. Characters such as “H” and “J”, which involve motion, require techniques like optical flow analysis or motion trajectory tracking using frame sequences. Integrating advanced models such as Long Short-Term Memory (LSTM) networks or 3D Convolutional Neural Networks (3D-CNNs) would enable the system to learn spatiotemporal dependencies, improving accuracy for motion-based signs. Additional improvements may include expanding the dataset to represent regional and cultural gesture variations and enhancing user interactivity through features such as real-time feedback, voice-guided instructions, and adaptive difficulty based on learner progress. Integration with educational platforms could further increase the application’s impact and accessibility.

Many sign languages include motion even in finger-spelled alphabets e.g., Chinese, Japanese, and Turkish systems. Future datasets should capture these nuanced gestures to support broader sign language recognition systems. Only a few countries, including the UK, Australia, and New Zealand, use dual-handed finger spelling, underscoring the importance of further research into symmetrical gesture interpretation. This study focused on static manual gestures, but future efforts should explore multimodal systems that incorporate facial cues and posture to provide a complete linguistic understanding. Large-scale datasets and deeper model architectures will be critical for achieving generalizable performance across diverse gesture types.

Punctuation, typically conveyed through non-manual markers like facial expressions or pauses, is currently outside the scope of this study. However, for more natural communication, future systems should integrate facial landmark detection, head tilt analysis, and timing models (e.g., RNNs or Transformers) to detect punctuation and prosodic features. These improvements would substantially enhance the depth, clarity, and naturalness of NZSL recognition systems [52].

This research also developed a working prototype capable of identifying and translating NZSL signs into English text. Looking ahead, a full-featured translation system could be created to support both text and speech output, accessible via mobile and web-based platforms. To explore deployment feasibility in educational settings, further evaluation involving teachers of Deaf students—both in specialized and mainstream classrooms should be undertaken. Moreover, a reverse-mode functionality could translate English text or speech into NZSL signs, expanding the app’s utility. Potential extensions include gesture-based control in gaming, educational interaction, and even robotic system interfaces, highlighting the system’s broad applicability across industries.

### 8.4. Conclusions

In conclusion, this project successfully developed a real-time recognition system tailored to New Zealand Sign Language, addressing a notable gap in gesture recognition technology. The model was trained on a purpose-built dataset of over 100,000 labeled hand landmark distances, achieving up to 99.52% accuracy using a Random Forest classifier. Through rigorous testing under varied environmental conditions, the system demonstrated high performance and resilience. The prototype integrates educational tools, enabling it to function as a learning aid while fostering digital inclusion.

This study has demonstrated that machine learning models, when combined with accurate and culturally specific datasets, can provide reliable sign language recognition. The system meets both technical benchmarks and user-centered design principles, including accessibility and ethical compliance. Overall, this project makes a measurable contribution to the growing field of inclusive communication technologies by offering a robust NZSL recognition tool with potential applications in education, translation, and assistive communication. Future work, focused on dynamic gesture modeling and multimodal input, will further improve system performance and broaden its societal impact.

## Figures and Tables

**Table 1:** Summary of Existing Related Work.

Study/Tech	Focus	Language	Key Contribution	Limitations
[36]	Gesture-based recognition system for NZSL	NZSL	Real-time translation of NZSL using dual-handed gestures.	Struggles with complex hand movements.
[37]	Machine learning for sign language recognition	General sign languages (includes NZSL)	Machine learning models for recognizing both one-handed and dual-handed signs.	Limited focus on dual-handed NZSL signs.
[38]	Evaluation of machine learning models for NZSL	NZSL	Highlights the gap in studies specific to NZSL.	No definitive conclusion on most effective algorithms.
[37]	Deep learning for gesture recognition	ASL, NZSL	Deep learning models for gesture recognition.	Limited to ASL; struggles with dual-handed NZSL signs.
General	Comparative analysis of sign language datasets	General sign languages	Compares available datasets for sign language recognition.	Insufficient dataset diversity for NZSL.

This table describes related literature view summary.

**Table 2:** Landmark and distances.

Hand 1	Hand 2	Description
unit-0	unit-28	Wrist to thumb base distance
unit-1	unit-29	Thumb base to middle distance
unit-2	unit-30	Middle to thumb tip distance
unit-3	unit-31	Thumb tip to index tip distance
unit-4	unit-32	Index tip to middle tip distance
unit-5	unit-33	Middle tip to ring tip distance
unit-6	unit-34	Ring tip to little tip distance
unit-7	unit-35	Little tip to palm base distance
unit-8	unit-36	Thumb base to index base distance
unit-9	unit-37	Index base to middle base distance
unit-10	unit-38	Middle base to ring base distance
unit-11	unit-39	Ring base to little base distance
unit-12	unit-40	Thumb base to index tip distance
unit-13	unit-41	Index base to middle tip distance
unit-14	unit-42	Middle base to ring tip distance
unit-15	unit-43	Ring base to little tip distance
unit-16	unit-44	Thumb base to middle tip distance
unit-17	unit-45	Thumb base to ring tip distance
unit-18	unit-46	Thumb base to little tip distance
unit-19	unit-47	Index base to ring tip distance
unit-20	unit-48	Index base to little tip distance
unit-21	unit-49	Middle base to little tip distance
unit-22	unit-50	Wrist to index tip distance
unit-23	unit-51	Wrist to middle tip distance
unit-24	unit-52	Wrist to ring tip distance
unit-25	unit-53	Wrist to little tip distance
unit-26	unit-54	Palm base to thumb-little midpoint distance
unit-27	unit-55	Palm base to index-little midpoint distance

This table describes landmark distances.

**Table 3:** Confusion Matrix for Actual and Predicted Values.

	Predicted True	Predicted False
**Actual True**	True Positive (TP)	False Negative (FN)
**Actual False**	False Positive (FP)	True Negative (TN)

**Table 4:** Overview of well-known sign language datasets and commonly used methods.

Dataset	Description	Common Methods Used	Data Type
**RWTH-PHOENIX-Weather 2014 (German Sign Language)**	Video-based dataset from weather broadcasts, widely used for continuous sign language recognition.	CNN–RNN hybrids (LSTM/GRU), Transformers, seq2seq with attention [47].	Video
**WLASL (Word-Level ASL)**	Large-scale ASL dataset with ∼21k samples at word level.	3D CNNs, I3D networks, Transformer architectures [48].	Video
**ASLLVD (American Sign Language Lexicon Video Dataset)**	Multi-view dataset for ASL lexical signs.	HMMs (early work), later CNN + RNN models for isolated sign recognition [49].	Video
**CSL (Chinese Sign Language)**	Several CSL datasets available, mostly video-based.	CNN + LSTM hybrids, other deep learning models for continuous/isolated recognition [50].	Video
**LSA64 (Argentinian Sign Language)**	Dataset of 64 isolated Argentinian signs.	CNN-based models for gesture classification [51].	Video
**NZSL (this work)**	Hand landmark distances (tabular, small-scale)	RF, SVM, kNN, etc.	Tabular

**Table 5:** Various Machine Learning Results.

Models	Accuracy	Precision	Recall	F1-Score
**Random Forest Classifier**	99.52%	99.53%	99.52%	99.52%
**K-Nearest Neighbour**	97.43%	97.44%	97.43%	97.43%
**Naïve Bayes**	96.96%	96.03%	96.97%	96.96%
**Decision Trees**	95.77%	95.78%	95.77%	95.77%
**Logistic Regression**	28.59%	20.20%	28.59%	21.24%
**Support Vector Machine**	22.13%	19.28%	22.13%	18.31%
**AdaBoost**	07.19%	03.30%	07.19%	03.73%

**Table 6:** Score of each alphabet and number.

Alphabet/Number	F1-Score	Alphabet/Number	F1-Score
1	1.00	6	0.99
2	1.00	7	0.99
3	1.00	8	0.99
4	0.99	9	1.00
5	1.00	10	1.00
A	1.00	L	1.00
B	0.99	M	1.00
C	0.99	N	1.00
D	1.00	O	1.00
E	0.99	P	1.00
F	0.99	Q	1.00
G	0.99	R	1.00
H	1.00	S	1.00
I	0.99	T	1.00
J	1.00	U	1.00
K	1.00	V	1.00
W	1.00	X	1.00
Y	1.00	Z	1.00

**Table 7:** Comparison with Previous Studies.

Feature	NZSL Research	YOLOv8	Arabic SL CNN	Source
Focus	Real-time NZSL alphabet detection using ML	Real-time hand detection for general gestures	Static Arabic sign recognition via CNN	Journal of AI, ML and Neural Network
Technologies Used	ML models (RF, KNN, SVM), OpenCV, Mediapipe	YOLOv8, OpenCV	DenseNet121, VGG16	TensorFlow, MediaPipe [14]
Gesture Type	NZSL alphabet (real-time)	General hand gestures (non-sign specific)	Static Arabic sign gestures	Arabic Sign Language Dataset
Dataset	100,000 NZSL gesture images	Dataset not specified	220,000 Arabic gesture images	Dataset details not specified
Model Type	Supervised ML classifiers (RF, KNN, SVM)	Deep learning with YOLOv8	Deep CNNs (DenseNet121, VGG16)	CNN-based multi-model
Real-Time Application	Yes, NZSL alphabet recognition	Yes, gesture detection and landmarking	No, focused on static gesture classification	Real-time sign language translation
Accuracy	99.52% with Random Forest	No explicit accuracy stated	100% in testing and validation	Not mentioned
Challenges Addressed	Real-time NZSL classification via ML	Eliminated manual features, improved detection performance	Accuracy boost for Arabic SL via multi-models	Communication gap bridging for deaf/mute communities
Key Innovation	Real-time NZSL for enhanced deaf/mute communication	YOLOv3-based general gesture recognition	Multi-model CNNs improving Arabic SL recognition	Multi-language support (ASL, ISL) for inclusivity

## Data Availability

The original contributions presented in the study are included in the article, further inquiries can be directed to the corresponding author.

## Abbreviations

The following abbreviations are used in this manuscript:

NZSL	New Zealand Sign Language
PSL	Pakistan Sign Language
BSL	British Sign Language
Auslan	Australian Sign Language
ASL	American Sign Language
CNN	Convolutional Neural Network
ML	Machine Learning
DL	Deep Learning
DT	Decision Tree
KNN	K-nearest neighbor
NB	Naive Bayes
SVM	Support Vector Machine
SIFT	Scale-Invarient Feature Transform
LR	Logistic Regression
RF	Random Forest
