# Correction: Ali et al. Real-Time Recognition of NZ Sign Language Alphabets by Optimal Use of Machine Learning. *Bioengineering* 2025, *12*, 1068

**DOI:** 10.3390/bioengineering13010068

**Published:** 2026-01-07

**Authors:** Mubashir Ali, Seyed Ebrahim Hosseini, Shahbaz Pervez, Muneer Ahmad

**Affiliations:** 1 Idexx Laboratories Inc., Auckland 4440, New Zealand; mubashir4ali@yahoo.com; 2 School of Information Technology, Whitecliffe College of Arts and Science, Auckland 1010, New Zealand; shahbazp@whitecliffe.ac.nz; 3 Department of Computer Science, University of Roehampton, London SW15 5PH, UK; muneer.ahmad@roehampton.ac.uk

Error in Author List.

In the original publication [1], the order of the authors was incorrect and listed as follows:

**Seyed Ebrahim Hosseini, Mubashir Ali, Shahbaz Pervez and Muneer Ahmad**

The correct order of the authors should be:

**Mubashir Ali, Seyed Ebrahim Hosseini, Shahbaz Pervez and Muneer Ahmad**

The authors have mistakenly added authors’ names in a different sequence.

The authors state that the scientific conclusions are unaffected. This correction was approved by the Academic Editor. The original publication has also been updated.
